# Learning and Acting in Peripersonal Space: Moving, Reaching, and Grasping

**DOI:** 10.3389/fnbot.2019.00004

**Published:** 2019-02-22

**Authors:** Jonathan Juett, Benjamin Kuipers

**Affiliations:** Computer Science and Engineering, University of Michigan, Ann Arbor, MI, United States

**Keywords:** sensorimotor learning, autonomous robot learning, peripersonal space, reaching and grasping, intrinsic motivation, developmental robotics

## Abstract

The young infant explores its body, its sensorimotor system, and the immediately accessible parts of its environment, over the course of a few months creating a model of peripersonal space useful for reaching and grasping objects around it. Drawing on constraints from the empirical literature on infant behavior, we present a preliminary computational model of this learning process, implemented and evaluated on a physical robot. The learning agent explores the relationship between the configuration space of the arm, sensing joint angles through proprioception, and its visual perceptions of the hand and grippers. The resulting knowledge is represented as the peripersonal space (PPS) graph, where nodes represent states of the arm, edges represent safe movements, and paths represent safe trajectories from one pose to another. In our model, the learning process is driven by a form of intrinsic motivation. When repeatedly performing an action, the agent learns the typical result, but also detects unusual outcomes, and is motivated to learn how to make those unusual results reliable. Arm motions typically leave the static background unchanged, but occasionally bump an object, changing its static position. The reach action is learned as a reliable way to bump and move a specified object in the environment. Similarly, once a reliable reach action is learned, it typically makes a quasi-static change in the environment, bumping an object from one static position to another. The unusual outcome is that the object is accidentally grasped (thanks to the innate Palmar reflex), and thereafter moves dynamically with the hand. Learning to make grasping reliable is more complex than for reaching, but we demonstrate significant progress. Our current results are steps toward autonomous sensorimotor learning of motion, reaching, and grasping in peripersonal space, based on unguided exploration and intrinsic motivation.

## 1. Introduction

### 1.1. What Is the Problem?

We observe that human infants are born without the ability to reach, grasp, and manipulate nearby objects. Their motions are seemingly aimless, but careful research has established that infants are biased toward moving objects and toward keeping the hands in view (von Hofsten, 1982, 1984; van der Meer et al., 1995; van der Meer, 1997). After a few months of unguided experience, human infants can reach deliberately to contact nearby objects, and after a few more months, they can grasp nearby objects with a reasonable degree of reliability (Berthier, 2011).

During the early process of learning to reach, children's arm trajectories are quite jerky, suggesting the underdamped behavior of partially tuned control laws (Thelen et al., 1993). A tempting hypothesis about early reaching is that visual servoing brings the images of the hand and the target object close together. However, an elegant experiment (Clifton et al., 1993) refutes this hypothesis by showing that young children's reaching behavior is unaffected when they can see the target object, but not their own hands. During later reach learning, children and adults move the arm and hand more smoothly and directly to the target object, and they start depending on visual access to the moving hand (Berthier, 2011).

**Figure 1:**
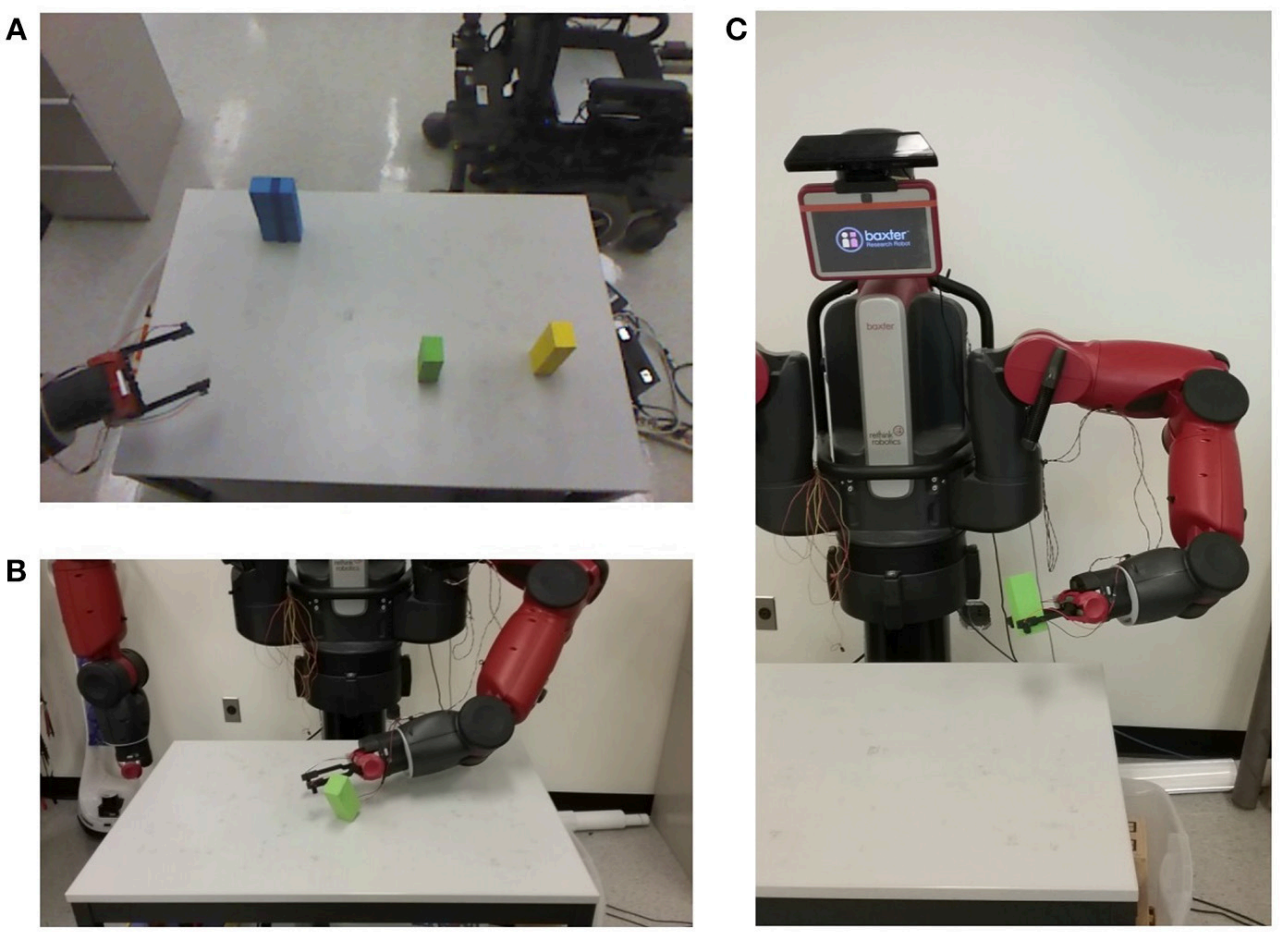
Our experiments are done using a Baxter robot from Rethink Robotics. **(A)** The environment from the agent's perspective, including simple block objects used in this work. **(B)** The unusual *bump* event is observed during random motions when the hand accidentally collides with an object and changes its state. The agent defines a *reach* action to make purposeful repetition of the bump event reliable. Actions are evaluated with a single foreground object present. **(C)** The *grasp* action allows the agent to control the motion of an object as it travels with the hand. The first grasps occur accidentally during reaching, and incorporation of additional preconditions and parameterizations makes intentional grasps increasingly reliable.

We abstract this developmental psychology problem to a problem in robot learning (Figure 1): How can a robot learn, from unguided exploratory experience, to reach and grasp nearby objects? We use the term *peripersonal space (PPS)* for the space immediately around the robot, accessible to its arms and hands for the manipulation of objects.

Peripersonal space includes multiple representations to accommodate different sensors and effectors. Proprioceptive sensors in the joints of the arm and hand provide information about the degrees of freedom of the manipulator, typically six or more. These degrees of freedom define the dimensions of the *configuration space*, in which a point determines the configuration of the arm, including the *pose* (position and orientation) of the hand. Vision provides sensory access to the 3D *workspace*, some but not all of which is within reach. To reach and grasp successfully, the robot needs to learn useful representations for the configuration space and the workspace, and for mappings between their different representations of peripersonal space.

Peripersonal space is also accessed by other sensory modalities such as touch and sound, and via other activities such as self-touch and tool use (Canzoneri et al., 2012, 2013; Roncone et al., 2016; Mannella et al., 2018). This paper focuses on learning from unguided exploration the functional relations linking proprioception and vision, two sensory modalities central to the representation of knowledge of peripersonal space. We hope to extend our approach to include touch and sound in the future.

### 1.2. Why Is the Problem Important?

Consider the computational problem faced by the newborn agent (human or robot), trying to make sense of the “blooming, buzzing confusion” of its sensory input, and learning to act with predictable and eventually useful results (Pierce and Kuipers, 1997). (Some of this learning could take place over evolutionary time, the learned knowledge being innate to the individual).

Reaching and grasping are among the earliest actions learned by a human infant, and they help it achieve control over its immediate environment, by being able to grasp an object, take control of it, and move it from one position to another. Reaching the desired object is a prerequisite for grasping. Moving the arm from one pose to another is a step toward learning to reach. All of this learning takes place through unguided exploration, without explicit instruction or reward.

From the early days of artificial intelligence, planners and problem-solvers (e.g., STRIPS, Fikes and Nilsson, 1971) assumed the existence of primitive actions for grasping and moving objects. This research contributes to showing how such primitives can be learned from very early experience.

### 1.3. Overview

A fundamental question about developmental learning is how an agent, without prior knowledge of its body, its sensors, its effectors, or its environment, can build a useful representation for the state of its world, and then can use this representation to learn reliable actions to change that state.

In our approach, the learning agent uses its unguided experience to define the peripersonal space (PPS) graph. Each node of the PPS graph represents a state of the arm, defined in terms of its joint angles, so it represents a point in configuration space. An edge linking two nodes is included when direct motion is safe between those two configurations. Each node is also annotated with the perceptual image(s) of the hand and arm in the otherwise empty workspace.

In this paper, we describe two applications of a general process for learning reliable actions. After creating the PPS graph, the process collects data about an initial action, learning its typical results, identifying unusual results, and then adding new preconditions or parameterizations to define a novel action that makes those unusual results reliable. We assume that a kind of intrinsic motivation (Baldassarre and Mirolli, 2013) drives this learning cycle. We use intrinsic motivation as a tool, but this paper is not intended as a contribution to the literature on intrinsic motivation.

The first application of the process observes the arm moving to configurations described by randomly-selected nodes in the PPS graph. The typical result is no change at all to the perceived images of blocks on the table; the main unusual result is a quasi-static change to the image due to the arm pushing or bumping (i.e., reaching) the block. Given a block to reach, the learning process finds preconditions that identify a target PPS node corresponding to that block, so that moving to that target node reliably reaches the intended block.

In the second application of the same process, the agent observes the result of reaching to randomly-selected blocks. Since the reach action is now reliable, the typical result is to cause a quasi-static change to the image of the selected block. The unusual result is for the block to move dynamically with the hand, rather than remaining static in a new position: the hand has grasped the block.

The conditions for making the grasp action reliable are more complex than for the reach action, but fortunately, they can still be expressed in terms of the PPS graph and the continuous spaces it approximates. Human infants, for several months after birth, exhibit the *Palmar reflex*, in which a touch on the palm causes the fingers to close tightly, automatically (and unintentionally) grasping an object (Futagi et al., 2012), the unusual event of an accidental grasp becomes frequent enough to provide sufficient data for a learning algorithm.

In this paper, we describe this process for learning increasingly reliable reach and grasp actions, without externally provided feedback or instruction. This paper improves, extends, and unifies results presented in our previous papers (Juett and Kuipers, 2016, 2018).

## 2. Related Work

### 2.1. The Human Model: Evidence From Child Development

There is a rich literature in developmental psychology on how infants learn to reach and grasp, in which the overall chronology of learning to reach is reasonably clear (e.g., Berthier, 2011; Corbetta et al., 2014). From birth to about 15 weeks, infants can respond to visual targets with “pre-reaching” movements that are generally not successful at making contact with the targets. From about 15 weeks to about 8 months, reaching movements become increasingly successful, but they are jerky with successive submovements, some of which may represent corrective submovements (von Hofsten, 1991), and some of which reflect underdamped oscillations on the way to an equilibrium point (Thelen et al., 1993). For decades, early reaching was generally believed to require visual perception of both the hand and the target object, with reaching taking place through a process of bringing the hand and object images together (“visual servoing”). However, a landmark experiment (Clifton et al., 1993) showed that the pattern and success rate of reaching by young infants is unaffected when the hand is not visible. Toward the end of the first year, vision of the hand becomes important for configuring and orienting the hand in anticipation of contact with target objects. The smoothness of reaching continues to improve over early years, toward adult reaches which typically consist of “a single motor command with inflight corrective movements as needed” (Berthier, 2011).

Theorists grapple with the problem that reaching and grasping require learning useful mappings between visual space (two- or three-dimensional) and the configuration space of the arm (with dimensionality equal to the number degrees of freedom).

Bremner et al. (2008) address this issue under the term, *multisensory integration*, focusing on sensory modalities including touch, proprioception, and vision. They propose two distinct neural mechanisms. The first assumes a fixed initial body posture and arm configuration, and represents the positions of objects within an egocentric frame of reference. The second is capable of re-mapping spatial relations in light of changes in body posture and arm configuration, and thus effectively encodes object position in a world-centered frame of reference.

Corbetta et al. (2014) focus directly on how the relation is learned between proprioception (“the feel of the arm”) and vision (“the sight of the object”) during reach learning. They describe three theories: vision first; proprioception first; and vision and proprioception together. Their experimental results weakly supported the proprioception-first theory, but all three had strengths and weaknesses.

Thomas et al. (2015) closely observed spontaneous self-touching behavior in infants during their first 6 months. Their analysis supports two separately-developing neural pathways, one for Reach, which moves the hand to contact the target object, and a second for Grasp, which shapes the hand to gain successful control of the object.

These and other investigators provide valuable insights into distinctions that contribute to answering this important question. But different distinctions from different investigators can leave us struggling to discern which differences are competing theories to be discriminated, and which are different but compatible aspects of a single more complex reality.

We believe that a theory of a behavior of interest (in this case, learning from unguided experience to reach and grasp) can be subjected to an additional demanding evaluation by working to define and implement a computational model capable of exhibiting the desired behavior. In addition to identifying important distinctions, this exercise ensures that the different parts of a complex theory can, in fact, work together to accomplish their goal.

The model we present at this point is preliminary. To implement it on a particular robot, certain aspects of the perceptual and motor system models will be specific to the robot, and not realistic for a human infant. To design, implement, debug, and improve a complex model, we focus on certain aspects of the model, while others remain over-simplified. For example, our model of the Peri-Personal Space (PPS) Graph uses vision during the creation of the PPS Graph, but then does not need vision of the hand while reaching to a visible object (Clifton et al., 1993). The early reaching trajectory will be quite jerky because of the granularity of the edges in the PPS Graph (von Hofsten, 1991), but another component of the jerkiness could well be due to underdamped dynamical control of the hand as it moves along each edge (Thelen et al., 1993), which is not yet incorporated into our model.

### 2.2. Robot Developmental Learning to Reach and Grasp

#### 2.2.1. Robotic Modeling

Some robotics researchers (e.g., Hersch et al., 2008; Sturm et al., 2008) focus on learning the kind of precise model of the robot that is used for traditional forward and inverse kinematics-based motion planning. Hersch et al. (2008) learn a body schema for a humanoid robot, modeled as a tree-structured hierarchy of frames of reference, assuming that the robot is given the topology of the network of joints and segments and that the robot can perceive and track the 3D position of each end-effector. Sturm et al. (2008) start with a pre-specified set of variables and a fully-connected Bayesian network model. The learning process uses visual images of the arm while motor babbling, exploiting visual markers that allow extraction of 6D pose for each joint. Bayesian inference eliminates unnecessary links and learns probability distributions over variable values. Our model makes weaker assumptions about the variables and constraints included in the model, and uses much weaker information from visual perception.

#### 2.2.2. Neural Modeling

Other researchers structure their models according to hypotheses about the neural control of reaching and grasping, with constraints represented by neural networks that are trained from experience. Oztop et al. (2004) draw on empirical data from the literature about human infants, to motivate their computational model (ILGM) of grasp learning. The model consists of neural networks representing the probability distributions of joint angle velocities. They evaluate the performance of their model with a simulated robot arm and hand, assuming that reaching is already programmed in. Their model includes a Palmar reflex, and they focus on learning an open-loop controller that is likely to terminate with a successful grasp.

Chinellato et al. (2011) propose an architecture consisting of two radial basis function networks linking retinotopic information with eye movements and arm movements through a shared head/body-centered representation. Network weights are trained through experience with a simulated 2D environment and 2 dof arm. Experiments demonstrate appropriate qualitative properties of the behavior.

Savastano and Nolfi (2013) describe an embodied computational model implemented as a recurrent neural network, and evaluated on a simulation of the iCub robot. They demonstrate pre-reaching, gross-reaching, and fine-reaching phases of learning and behavior, qualitatively matching observations of children such as diminished use of vision in the first two phases, and proximal-then-distal use of the arm's degrees of freedom. The transitions from one phase to the next are represented by manually adding certain links and changing certain parameters in the network, begging the question about how and why those changes take place during development.

Caligiore et al. (2014) present a computational model of reach learning based on reinforcement learning, equilibrium point control, and minimizing the speed of the hand at contact. The model is implemented on a simulated planar 2 dof arm. Model predictions are compared with longitudinal observations of infant reaching between ages of 100 and 600 days (Berthier and Keen, 2006), demonstrating qualitative similarities between their predictions and the experimental data in the evolution of performance variables over developmental time. Their focus is on the irregular, jerky trajectories of early reaching (Berthier, 2011), and they attribute this to sensor and process noise, corrective motions, and underdamped dynamics (Thelen et al., 1993). By contrast, we attribute part of the irregular motion to the irregularity of motion along paths in the PPS graph (rather than to real-time detection and correction of errors in the trajectory, which would be inconsistent with Clifton et al., 1993). We accept that other parts of this irregularity is likely due to process noise and underdamped dynamics during motion along individual edges in the PPS graph, but that aspect of our model is not yet implemented. At the same time, the graph representation we use to represent early knowledge of peripersonal space can handle a realistic number of degrees of freedom in a humanoid robot manipulator (Figure 1).

#### 2.2.3. Sensorimotor Learning

Several recent research results are closer to our approach, in the sense of focusing on sensorimotor learning without explicit skill programming, exploration guidance, or labeled training examples. Each of these (including ours) makes simplifying assumptions to support progress at the current state of the art, but each contributes a “piece of the puzzle” for learning to reach and grasp.

Our work is closely related to the developmental robotics results of Law et al. (2014a,b). As in their work, we learn graph-structured mappings between proprioceptive and visual sensors, and thus between the corresponding configuration space and work space. Like them, we apply a form of intrinsic motivation to focus the learning agent's attention on unusual events, attempting to make the outcomes reliable. A significant difference is that Law et al. (2014a,b) provide as input an explicit schedule of “constraint release” times, designed to follow the observed stages identified in the developmental psychology literature. Our goal is for the developmental sequence to emerge from the learning process as pre-requisite actions (e.g., reaching) must be learned before actions that use them (e.g., grasping).

Jamone et al. (2012, 2014) define a Reachable Space Map over gaze coordinates (head yaw and pitch, plus eye vergence to encode depth) during fixation. The control system moves the head and eyes to place the target object at the center of both camera images. Aspects of this relationship between retinal, gaze, and reach spaces were previously investigated by Hülse et al. (2010). In the Reachable Space Map, *R* = 0 describes unreachable targets; intermediate values describe how close manipulator joints are to the physical limits of their ranges; and *R* = 1 means that all joints are well away from their limits. The Reachable Space Map is learned from goal-directed reaching experience trying to find optimal reaches to targets in gaze coordinates. Intermediate values of *R* can then be used as error values to drive other body-pose degrees of freedom (e.g., waist, legs) to improve the reachability of target objects. Within our framework, the Reachable Space Map would be a valuable addition (in future work), but the PPS Graph (Juett and Kuipers, 2016) is learned at a developmentally earlier stage of knowledge, before goal-directed reaching has a meaningful chance of success. The PPS Graph is learned during non-goal-directed motor babbling, as a sampled exploration of configuration space, accumulating associations between the joint angles determining the arm configuration and the visual image of the arm.

Ugur et al. (2015) demonstrate autonomous learning of behavioral primitives and object affordances, leading up to imitation learning of complex actions. However, they start with the assumption that peripersonal space can be modeled as a 3D Euclidean space, and that hand motions can be specified via starting, midpoint, and endpoint coordinates in that 3D space. Our agent starts with only the raw proprioceptively sensed joint angles in the arm and the 2D images provided by vision sensors. The PPS graph represents a learned mapping between those spaces. The egocentric Reachable Space Map (Jamone et al., 2014) could be a step toward a 3D model of peripersonal space.

Hoffmann et al. (2017) integrate empirical data from infant experiments with computational modeling on the physical iCub robot. Their model includes haptic and proprioceptive sensing, but not vision. They model the processes by which infants learn to reach to different parts of their bodies, prompted by buzzers on the skin. They report results from experiments with infants, and derive constraints on their computational model. The model is implemented and evaluated on an iCub robot with artificial tactile-sensing skin. However, the authors themselves describe their success as partial, observing that the empirical data, conceptual framework, and robotic modeling are quite disparate, and not well integrated. They aspire to implement a version of the sensorimotor account, but they describe their actual model as much closer to traditional robot programming.

## 3. Building the Peripersonal Space Graph

### 3.1. Methods

A baby begins to explore its environment and the range of motion of its arms with seemingly random movements and no clear external goal.

There is a physical relationship between the configuration **q** of the arm in configuration space, and the resulting pose **p** of the hand in the workspace. This relationship, *forward kinematics*, is not known to the baby.

(1) $$\begin{array}{r} f\left(\text{q}\right)=\text{p} \end{array}$$

The physical structure of the robot and its perceptual system also define a mapping from the pose of the hand to a visual representation (e.g., a binary image) of the hand. (Note that *I*_*p*_ is simply an identifier for an image, and does not allow the agent to obtain an explicit representation of the pose **p**).

(2) $$\begin{array}{r} I\left(\text{p}\right)=I_{p} \end{array}$$

Composing these defines a (partial) function *g* that the robot *can* learn about, by simultaneously using proprioception to sense the configuration **q**, and visual perception to sense the image *I*_*p*_.

(3) $$\begin{array}{r} g\left(\text{q}\right)=I\left(f\left(\text{q}\right)\right)=I_{p} \end{array}$$

This observation (**q**, *I*_*p*_) is one point on the function *g*.

The Peripersonal Space (PPS) graph P is a collection of nodes and edges, representing a state of knowledge about the mapping *g*.^1^ A node *n* ∈ P represents an observation (**q**, *I*_*p*_). An edge (*n*_*i*_, *n*_*j*_) = *e*_*ij*_ ∈ P represents an *affordance* (i.e., an opportunity) for safe motion between **q**(*n*_*i*_) and **q**(*n*_*j*_).

The robot learning agent creates a PPS graph P of *N* nodes by sampling the configuration space of its arm. From an initial pose **q**_0_ in an empty environment, the robot samples a sequence of perturbations Δ**q** from a distribution D to generate a sequence of poses:

(4) $$\begin{array}{r} {\text{q}}_{i+1}={\text{q}}_{i}+\Delta {\text{q}}_{i}\text{while }i\in \left[0,N-1\right] \end{array}$$

While the motor babbling of human infants may appear random, it does exhibit biases toward moving objects and toward keeping the hand visible (von Hofsten, 1982, 1984; van der Meer et al., 1995; van der Meer, 1997). We use *rejection sampling* to enforce these biases, and constraints against collisions with the table or the robot's own body. If either condition is violated, the proposed configuration is rejected and a new *q*_*i*+1_ is sampled.

At this point, the arm is physically moved from its current configuration **q**_1_ to the new configuration **q**_*i*+1_. After each new pose has been safely reached by physical motion of the arm, a corresponding perceptual image *I*_*p,i*+1_ is collected, and the node *n*_*i*+1_ = (**q**_*i*+1_, *I*_*p,i*+1_) and the undirected edge *e*_*i, i*+1_ = (*n*_*i*_, *n*_*i*+1_) are added to P. The length of an edge is the Euclidean distance between the configurations at its endpoint nodes, considered in joint space.

(5) $$\begin{array}{r} ||e_{ij}||=d\left(n_{i},n_{j}\right)=||{\text{q}}_{i}-{\text{q}}_{j}|{|}_{2} \end{array}$$

At this point, the graph is a linear chain, so between any two nodes there is a single path, typically very long. In addition to inefficiency, having a single path through the graph does not provide options for avoiding obstacles or selecting the most reliable approach for a learned action. The graph needs much higher connectivity, by adding new edges linking existing nodes in P.

It is not feasible to test every pair of unconnected nodes, so we apply a heuristic. Let the length of an edge be the Euclidean distance between the configurations at its endpoint nodes, considered in joint space.

(6) $$\begin{array}{r} ||e_{ij}||=d\left(n_{i},n_{j}\right)=||{\text{q}}_{i}-{\text{q}}_{j}|{|}_{2} \end{array}$$

and let μ_*e*_ be the mean length of all the edges in the current (linear) graph. The heuristic is that when *d*(*n*_*i*_, *n*_*j*_) < μ_*e*_, the average length of edges known from exploration to be safe, then the edge *e*_*ij*_ can be added to P, if it is not already present. With the inclusion of these edges, we expect that P will supports planning of multiple trajectories between any given pair of nodes. Because P is still a sparse approximation to the configuration space, trajectories across the environment will tend to be jerky.

Any path 〈*n*_1_, …, *n*_*m*_〉 in a PPS graph P corresponds with a safe trajectory 〈**q**_1_, …, **q**_*m*_〉 of the arm. The agent designates a *home node*, *n*_*h*_, where the arm rests naturally and that allows relatively unoccluded observation of the environment. By convention, trajectories begin at *n*_*h*_, and eventually return there, too. We will also define the terms *n*_*f*_ for the final node of a trajectory, and *n*_*p*_ for the penultimate node.

The PPS graph P can then be used as “scaffolding” to learn increasingly expert ways to reach and grasp. By searching the information in the PPS graph P, we can define a function *h* that provides a discrete approximation to *g*^−1^ from Equation (3):

(7) $$\begin{array}{r} C\left(I_{b}\right)=\left\{\left(\text{q},I_{p}\right)=n\in P:match\left(I_{b},I_{p}\right)\right\} \end{array}$$

(8) $$\begin{array}{r} h\left(I_{b}\right)={\text{q}}^{*}=select_{\text{q}}C\left(I_{b}\right) \end{array}$$

Given a current visual image *I*_*b*_ of an object (e.g., a block) in the environment, we can identify nodes (**q**, *I*_*p*_) = *n* ∈ P whose stored images *I*_*p*_ of the hand matches (e.g., overlaps with) the currently sensed image *I*_*b*_ of the object. The generic operator *select*_**q**_ defines the role for a criterion for selecting among matching nodes, for example by maximizing the overlap between binary images *I*_*b*_ and *I*_*p*_, or by minimizing the distance between their centers.

### 3.2. Experiment 1: Creating the Peripersonal Space Graph

For our experiment, we apply the methods described above (section 3.1) to learn to control the left arm of our Baxter Research Robot (Figure 1), providing specific instantiations for the generic aspects of the method. The state of this arm can be given by eight degrees of freedom, a set of seven joint angles, $\text{q}=\langle q^{1},\ldots ,q^{7}\rangle =\langle s_{0},s_{1},e_{0},e_{1},w_{0},w_{1},w_{2}\rangle$ and the aperture *a* between the gripper fingers, described by a percentage of its maximum width.

**Figure 2:**
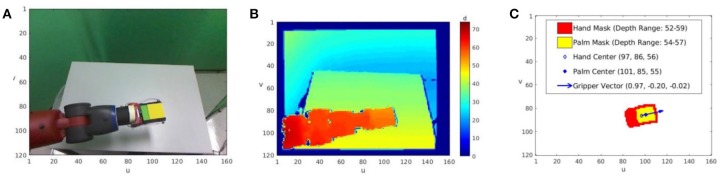
An example of the agent's visual percept and stored representation for a node *n*_*i*_. **(A)** A single RGB image *I*_*RGB*_, scaled down to 120 × 160 resolution, taken while the arm configuration is set to q_*i*_ = q(*n*_*i*_). **(B)** The registered depth image *I*_*D*_ taken at the same time. Note that the depth values are a measure of disparity, so smaller values are further from the camera. **(C)** The full representation the agent stores for the node *n*_*i*_. Aided by the yellow block held between the gripper fingers, the agent segments the palm mask, corresponding to the grasping region of the hand. The larger hand mask includes the palm mask (shown in yellow) and parts of the robot image segment near the block, typically the gripper fingers and lower wrist (shown in red). The range of depth image values within each mask is also stored, as are the center of mass and mean depth value for each mask. Finally, to estimate the direction the grippers are pointing, a vector is drawn from the hand mask center through the palm mask center.

For the Baxter Research Robot, each visual percept *I*_*p*_ is taken by a fixed-viewpoint RGB-D camera, providing an RGB image *I*_*RGB*_ and a depth-registered image *I*_*D*_ (Figure 2). During the construction of P, the agent may save a percept *P*(*n*_*i*_) taken while it is paused at *n*_*i*_.

For our experiment, the robot begins with an empty PPS Graph P, and the arm is initially at the home configuration **q**_*h*_ = **q**(*n*_*h*_). The random motor babbling search described in Equation (4) is instantiated for our robot in a straight-forward way. For each joint angle *k*, the displacement to add is sampled from a normal distribution with a standard deviation equal to a tenth of the full range of that joint.

(9) $$\begin{array}{r} q_{i+1}^{k}=q_{i}^{k}+\Delta q^{k}\text{where}\Delta q^{k}\sim N\left(0,\sigma_{k}\right)\text{and}\sigma_{k}=0.1\cdot range\left(q^{k}\right) \end{array}$$

We impose a bias using a form of *rejection sampling*, requiring that the resulting end-effector pose must fall within the field of view, and must not collide either with the table or with the robot's own body. If either condition is violated, the proposed configuration is rejected and a new *q*_*i*+1_ is sampled. As noted previously, human infants exhibit a bias toward keeping the hand visible (von Hofsten, 1982, 1984; van der Meer et al., 1995; van der Meer, 1997). Human infants are also soft and robust, so they can detect and avoid collisions with minimal damage. To prevent damage to our Baxter Research Robot, we implement these checks using a manufacturer-provided forward kinematics model that is below the level of detail of our model, and is used nowhere else in its implementation. In future work, we will considering biasing this sampling to resemble human infants' pre-reaching motions toward objects, or to move in a cyclic fashion, often returning to the center of the field of view.

To move along an edge *e*_*ij*_ from *n*_*i*_ to *n*_*j*_, in the current implementation, the agent uses linear interpolation of each joint angle *q*^*k*^ from its value in **q**_*i*_ to its value in **q**_*j*_.

For this experiment, the total number of nodes created and added to P is *N* = 3, 000.

### 3.3. Experiment 1 Results

**Figure 3:**
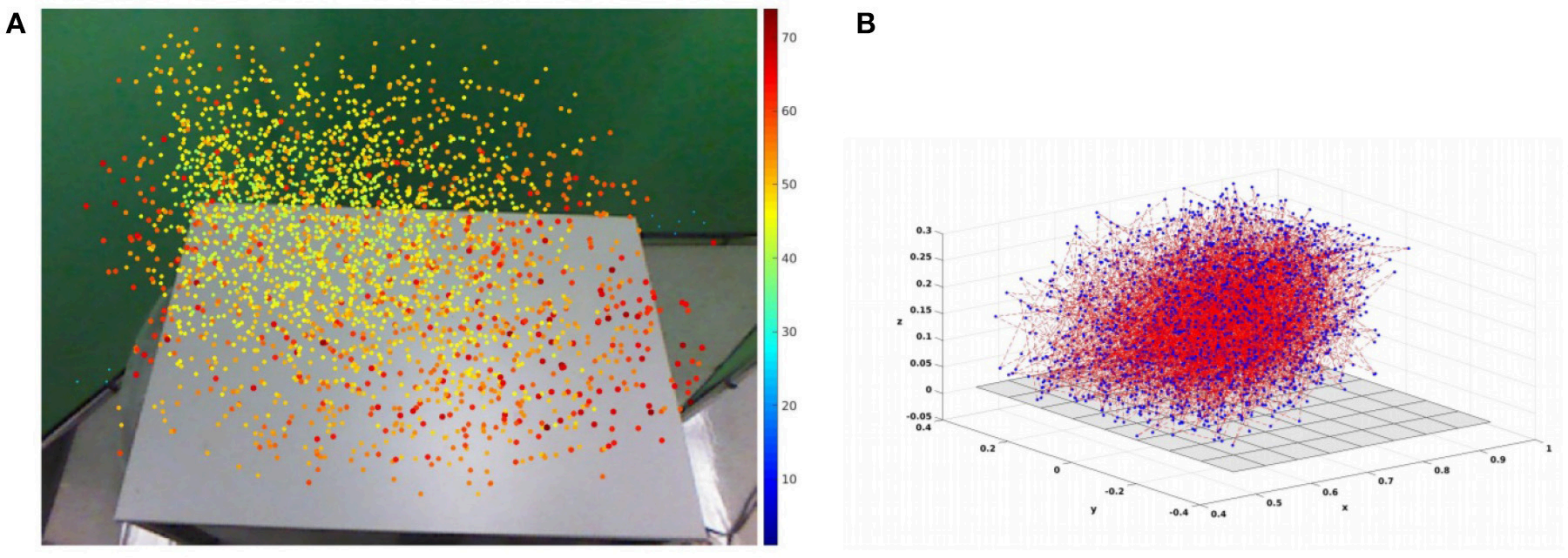
Two visualizations of the Peripersonal Space (PPS) Graph P, with size *N* = 3, 000. Each visualization shows wide coverage that facilitates movement throughout the environment, with a few sparse patches. **(A)** An example RGB percept of the empty environment, overlayed with the (*u, v*) center of mass locations for all *N* nodes. The dot for each node is colored and sized according to its mean disparity value *d* (see key along right edge). Nodes with higher disparity (closer to the camera) appear larger and more red, while nodes with lower disparity (farther from the camera) appear smaller and closer to blue. **(B)** The nodes of P displayed in the true world (*x, y, z*) coordinates of the Baxter Robot's default frame of reference. The gray plane represents the surface of the table. Nodes are plotted as blue points. The 2,999 edges in the original chain from motor babbling are shown as dotted red lines. Not shown are the edges added according to the safe motion heuristic.

The Peripersonal Space graph P is a sparse approximation of the configuration space of the robot arm (Figure 3). It is evident that random sampling through unguided exploration has distributed *N* = 3, 000 nodes reasonably well throughout the workspace, with some localized sparse patches and a region in the far right corner that is generally out of reach of the robot's left hand. The display in Figure 3A overlays information available to the robot in the individual nodes of P. The information in Figure 3B is not available to the robot.

Random exploration of the configuration space with *N* = 3, 000 creates 3,000 nodes, in a chain with 2,999 edges. Of the original 2,999 edges, 1,614 of them have length less than the mean length μ_*e*_ of all 2,999 edges. The heuristic that creates a new edge between *n*_*i*_ and *n*_*j*_ when *d*(*n*_*i*_, *n*_*j*_) < μ_*e*_ adds 108,718 new edges, so that P now has 3,000 nodes and 111,717 edges. By comparison, the complete graph with 3,000 nodes has 4,448,500 edges, so the PPS graph P has the same number of nodes and about 2% as many edges as the complete graph.

## 4. Learning a Reliable Reach Action

In our model, learning the reach action takes place in three stages. First, the agent must learn to detect the unusual event of bumping a block, causing a quasi-static change in the environment, against the background of typical arm motions that leave the environment unchanged. Second, the agent learns criteria for selecting nodes from the PPS graph, such that moving to one of those nodes increases the likelihood of bumping a specified block. Third, the agent learns how to interpolate in continuous space between the nodes of the PPS graph to further increase the likelihood of bumping a target block.

Since these three learning stages have different character, depend on different knowledge, and apply different methods, we describe our research on each of them with its own Methods-Experiments-Results description.

### 4.1. Observing the Unusual Event of a Bump

#### 4.1.1. Methods

During the construction of the PPS Graph, the agent's perceptual input can be easily factored into a static background, and a highly variable foreground corresponding to the robot's hand and arm. This allows the nodes of the PPS Graph to be characterized by the perceptual image of the robot's hand. By detecting a correlation between “random” motor output and perceived hand motion, the agent can diagnose that that the hand is part of the agent's “self.”

Once the PPS Graph has been completed, additional objects are placed into the workspace. The objects used for this work are rectangular prism blocks with a single long dimension. The blocks are placed upright at randomly generated coordinates on the table in front of the robot, with the requirement that each placement leaves all blocks unoccluded and fully within the field of vision. The objects have distinctive colors not present in the background, making it easy to create a binary image mask for each object in the RGB image. This image mask can be applied to the depth image to determine the range of depth values associated with the object.

The agent creates binary image masks as more efficient representations of its own hand and of foreground objects that may be targets of actions. For each *n*_*i*_ ∈ P, the agent finds the end effector in *I*_*RGB*_(*n*_*i*_) and records two binary masks that describe its location in the image. The *palm mask p*_*i*_ is defined to be the region between the gripper fingers, which will be most relevant for grasping.^2^ The *hand mask h*_*i*_ includes this region as well as the gripper fingers and the wrist near the base of the hand. *h*_*i*_ reflects the full space occupied by the hand, which is most useful to identify and avoid nodes with hand positions that may collide with obstacles. The state representation for a node also includes the range of depths the end effector is observed to occupy. This range is found by indexing into *I*_*D*_(*n*_*i*_) with either mask, and determining the minimum and maximum depth values over these pixels. That is, the depth range of the palm *D*(*p*_*i*_) ≡ [min(*I*_*D*_(*n*_*i*_)[*p*_*i*_]), max(*I*_*D*_(*n*_*i*_)[*p*_*i*_])], and the depth range of the full hand *D*(*h*_*i*_) is defined analogously. Edges can also be associated with a binary mask for the area swept through during motion along it, $s_{i,i^{\prime }}$, approximated by a convex hull of the hand masks of the endpoint nodes, *h*_*i*_ and $h_{i^{\prime }}$. The depth range of motion along an edge is the full range between the minimum and maximum depths seen at either endpoint, $D(s_{i,i^{\prime }})\equiv [\text{min}(D(h_{i}),D(h_{i^{\prime }})),\text{ max}(D(h_{i}),D(h_{i^{\prime }})]$.

Many (but not all) motions of the arm leave the other objects unaffected, so the new objects typically behave as part of the static background model. However, occasionally the hand bumps into one of the objects and knocks it over or shifts its position. This is defined as a *bump* event, and is detected by the agent as a quasi-static change to the perceptual image of the object.

When an image of an object is characterized by a binary mask, the difference between two images *A* and *B* can be measured by the *Intersection Over Union* measure:

(10) $$\begin{array}{r} IOU\left(A,B\right)=|A\cap B|/|A\cup B|. \end{array}$$

Comparing the images of an object *A* at times *t*_1_ and *t*_2_, when *IOU*(*A*(*t*_1_), *A*(*t*_2_)) ≈ 1 the object has remained static. In case we observe *IOU*(*A*(*t*_1_), *A*(*t*_2_)) ≪ 1, the object may have moved, but we take care to exclude the case of a temporary occlusion of an object by the hand or arm.

We define a *reach* as the action of following a trajectory resulting in a bump event with a target object. Even without knowing how to make a *reach* action reliable, the *IOU* criterion will allow the agent itself to distinguish between successful and unsuccessful *reach* actions. In subsequent stages, the agent will learn how to *reach* reliably.

#### 4.1.2. Experiments

The agent continues to practice its new capability to perform motions allowed by the PPS Graph and observe the results of these motions.

##### 4.1.2.1. Experiment 2: Exploration

The agent follows this procedure:

1. Observe the environment while at the home node *n*_*h*_, and find the initial mask for each of three objects newly placed in the foreground.
2. Select a random final node *n*_*f*_ in the PPS Graph.
3. Perform a graph search to determine the shortest path trajectory from the home node *n*_*h*_ to *n*_*f*_.
4. Execute the trajectory, checking the visual percept at each node for any significant change to an object mask.
5. If a change is observed, or the current node is *n*_*f*_, immediately return to the home node along the shortest path.
6. Calculate the *IOU* values between the initial and final masks for each object.
  - If an apparent change at intermediate node *n*_*i*_ that triggered an immediate return is not confirmed (i.e., *IOU* ≈ 1), then repeat the trajectory, continuing past *n*_*i*_, to search for a subsequent bump event.
7. Cluster all *IOU* values seen so far into two clusters.
8. Repeat until the smaller cluster contains at least 20 examples.

By clustering the results of the *IOU* criterion, the agent learns to discriminate between the typical outcome of a trajectory (no change) and an unusual outcome (a bump event). These outcomes are defined as the unsuccessful and successful results of a *reach* action, respectively. Subsequent stages will identify features to allow increasingly reliable *reach* actions.

##### 4.1.2.2. Experiment 3: Reach reliability

To quantify this improvement, we establish a baseline level of performance for the policy of selecting a random final node *n*_*f*_ and then following the shortest path in the PPS Graph to *n*_*f*_. This second experiment consists of 40 trials with a single, randomly-placed target block.

#### 4.1.3. Results

Following this procedure, with three new objects added to the environment, the agent moved along 102 trajectories and gathered 306 IOU values between initial and final object masks. Where *t* is the target object mask prior to the motion, and *t*′ is the target object mask following the motion, the *IOU* values fell into two well-separated clusters.

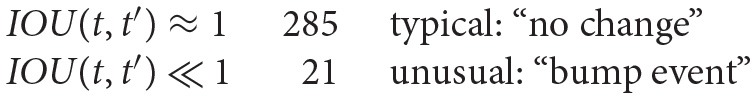



Intuitively, a trajectory to a random final node is unlikely to interact with an object on the table. However, in a rare event the hand bumps the object, knocking it over, or sliding it along the table and sometimes off the table (the resulting absence of a final mask leads to an IOU of 0, so no special case is necessary).

The strategy of returning to the home node to observe the final mask allows the agent to rule out occlusion by the hand as the source of the perceptual change. This has not been observed to make false positive bump classifications. This is important so that the agent will not learn incorrect conditions for a bump. There are a small number of false negatives where the hand and object do collide, but without lowering the IOU enough to fall into the smaller cluster. The agent is still able to learn the conditions from the reduced number of observed bumps, and may even favor actions that cause larger, more reliable bumps as a result.

The agent can classify all future motions in the presence of an object by associating the resulting observed IOU with one of the two clusters. While we human observers can describe the smaller cluster as a *bump* event, the robot learning agent knows only that the smaller cluster represents an unusual but recognizable event, worth further exploration. The agent has no knowledge of what makes a reach succeed. The following stages will help fill that gap.

The quantitative baseline experiment gives a reliability of 20% for the reach action to a random final node, which will be compared other methods in **Figure 7**.

**Table:** 

**Reach reliability given selection method for *n*_*f*_**	
Select random target node *n*_*f*_ from PPS graph (baseline)	20.0%

### 4.2. Identifying Candidate Final Nodes

#### 4.2.1. Methods

The agent has identified the rare event of a *bump*, and has defined *reach* as the action that can cause this event. Choosing a target node *n*_*f*_ randomly from the PPS graph gives a baseline reliability of 20%. The agent is now intrinsically motivated to search for ways to improve the reliability of the *reach* action. This can be done by identifying one or more features that discriminate between the cases that result in a bump, and those that do not.

The PPS graph stores a visual percept of the hand on each node, and the agent has a current visual percept of the target object. Comparing these percepts is straightforward, since they have the same frame of reference, and the agent has the RGB masks and the depth ranges from each image. Any nonempty intersection predicts that the hand and the target object will occupy the same region of the RGB image, or the same depth, or both.

The stored visual percepts also allow the agent to derive the image-space center of mass of the end effector at a given node. Centers and directions will have three components, two for the (*u, v*)-coordinates in the RGB image, and one (*d*) for depth values in the Depth image. For a node *n*_*i*_, the center of the palm $c_{i}^{p}$ is composed of the center of mass of *p*_*i*_ and the average depth, mean(*P*_*D*_(*n*_*i*_)[*p*_*i*_]), and the center of the hand $c_{i}^{h}$ is derived from *h*_*i*_ and *P*_*D*_(*n*_*i*_)[*p*_*i*_] in the same manner. Center *c*^*t*^ for a target object with mask *t* and depth range *D*(*t*) in the current percept is also found analogously.

Using the PPS graph, the agent improves *reach* reliability in three steps.

1. Determine which binary image masks and which intersection property best predict the occurrence of a *bump* event.
2. Identify a set of candidate final nodes from the PPS graph with this intersection property. Select an arbitrary node in this set as the target node *n*_*f*_.
3. Determine the best measure of closeness between centers of palm and target object, and select the closest node *n*_*f*_ from the candidate final node set.

#### 4.2.2. Experiments

##### 4.2.2.1. Experiment 4: Which intersection property is best?

By further analysis of the data reported in section 4.1.3 from 102 reaching trajectories, the agent can determine which binary image mask, and which intersection property, best predict whether a trajectory will produce a *bump* event.

The agent compares binary masks *b* representing the palm (*p*_*f*_) or the hand (*h*_*f*_) at its final pose or throughout its final motion (*s*_*p,f*_). For each binary mask *b* and the mask *t* representing the target object, the trajectories are placed in four groups according to whether *b* ∩ *t* and/or *D*(*b*) ∩ *D*(*t*) are empty or nonempty. Counts of observed bumps and the total number of trajectories within each group allow the conditional probabilities of a bump to be computed.

The set of PPS graph nodes that satisfy the selected mask intersection property, with the best choice of mask, will define the set of *candidate final nodes* for a *reach* trajectory.

##### 4.2.2.2. Experiment 5: Using the candidate final nodes

An improved reach action policy can be created by selecting the target node *n*_*f*_ as a random member of the candidate final node set, rather than a random node from the entire PPS graph. The shortest graph path is found from the home node *n*_*h*_ to this final node *n*_*f*_. This policy is evaluated using the same method as Experiment 2 in section 4.1.2: reaching for 40 blocks, presented individually at randomly assigned locations on the table.

##### 4.2.2.3. Experiment 6: Selecting the best candidate node

In spite of every candidate node having non-empty intersections between both RGB and D masks of hand and target object, the reliability of this reach action is still only 52.5%. One reason is that the RGB and D masks taken together over-estimate the space occupied by the hand or an object, so the intersection may take place in empty space. Another reason is that some non-empty intersections may be very small, resulting in an imperceptible bump event.

To address this issue, we identify a distance measure between hand and target object, and then select from the set of candidate nodes, the node that minimizes that distance measure. Once this node is chosen, the rest of the path is planned as before. This improved policy is evaluated the same way as Experiment 4.

#### 4.2.3. Results

##### 4.2.3.1. Experiment 4 results

The set of groups where *b* = *p*_*f*_ contains the group with the highest conditional probability.

Each array represents the four possible intersection conditions, and each entry holds the conditional probability of a *bump* event in a trajectory satisfying that intersection conditioned, explained as the ratio of *bump* events to trajectories. Recall that three objects were present for each trajectory, so the total number of observations reflected in the denominators is 306.

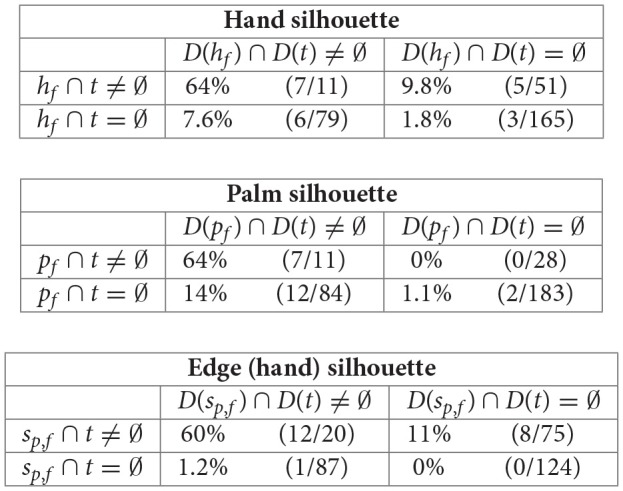



A bump is most likely (64%) to occur at a final node *n*_*f*_ where the palm percept has a nonempty intersection in both mask and depth range with the target percept, that is, where

(11) $$\begin{array}{r} p_{f}\cap t\neq \emptyset \wedge D\left(p_{f}\right)\cap D\left(t\right)\neq \emptyset . \end{array}$$

**Figure 4:**
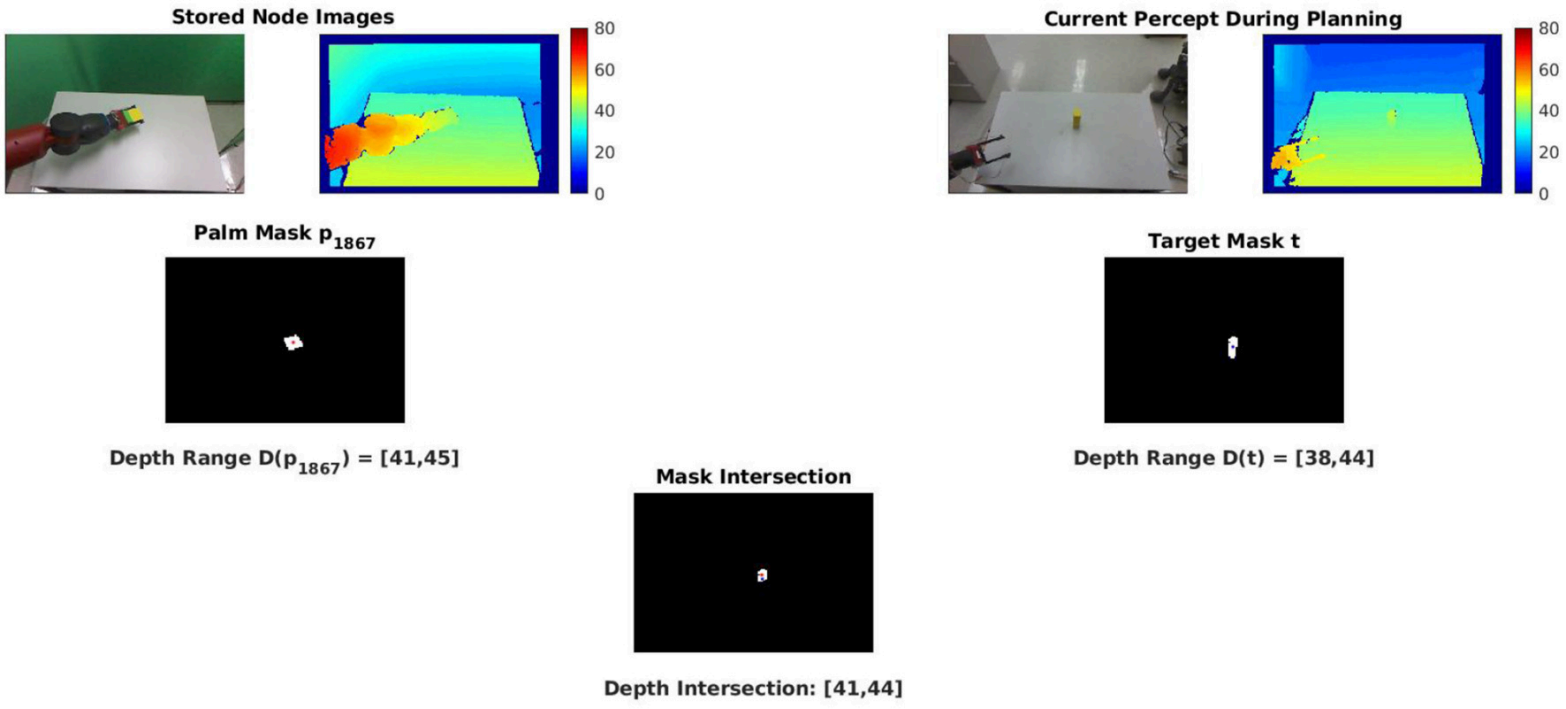
Candidate final nodes are identified from their intersection features. **(Top row)** RGB-D percepts taken from the node definition (left two) and from the current percept of the environment (right two). **(Middle row)** The palm masks and depth ranges from the stored percept (left) and current percept (right). **(Bottom row)** The intersections of the palm masks and the depth ranges are both non-empty, so the current node is identified as a candidate for reaching the observed block. The palm masks and depth ranges for each node can be computed in advance. The intersections of the mask and range from a target block can be quickly evaluated for all 3,000 PPS graph nodes to generate the set of candidate final nodes.

The process of identifying a node as a candidate is demonstrated in Figure 4.

##### 4.2.3.2. Experiment 5 results

For the same 40 placements as the baseline (Experiment 2), 39 have at least one node with both mask and depth range intersections with the target (i.e., has a non-empty candidate final node set), and the policy of moving to one of these nodes bumps the target 21 times. Attempting a reach to the placement where no node has both RGB and Depth intersections was not successful. Overall, the reach action is now 52.5% reliable. The comparison in **Figure 7** shows reaching to an arbitrary candidate node is more than twice as reliable as the baseline action of moving to a random final node.

##### 4.2.3.3. Experiment 6 results

**Figure 5:**
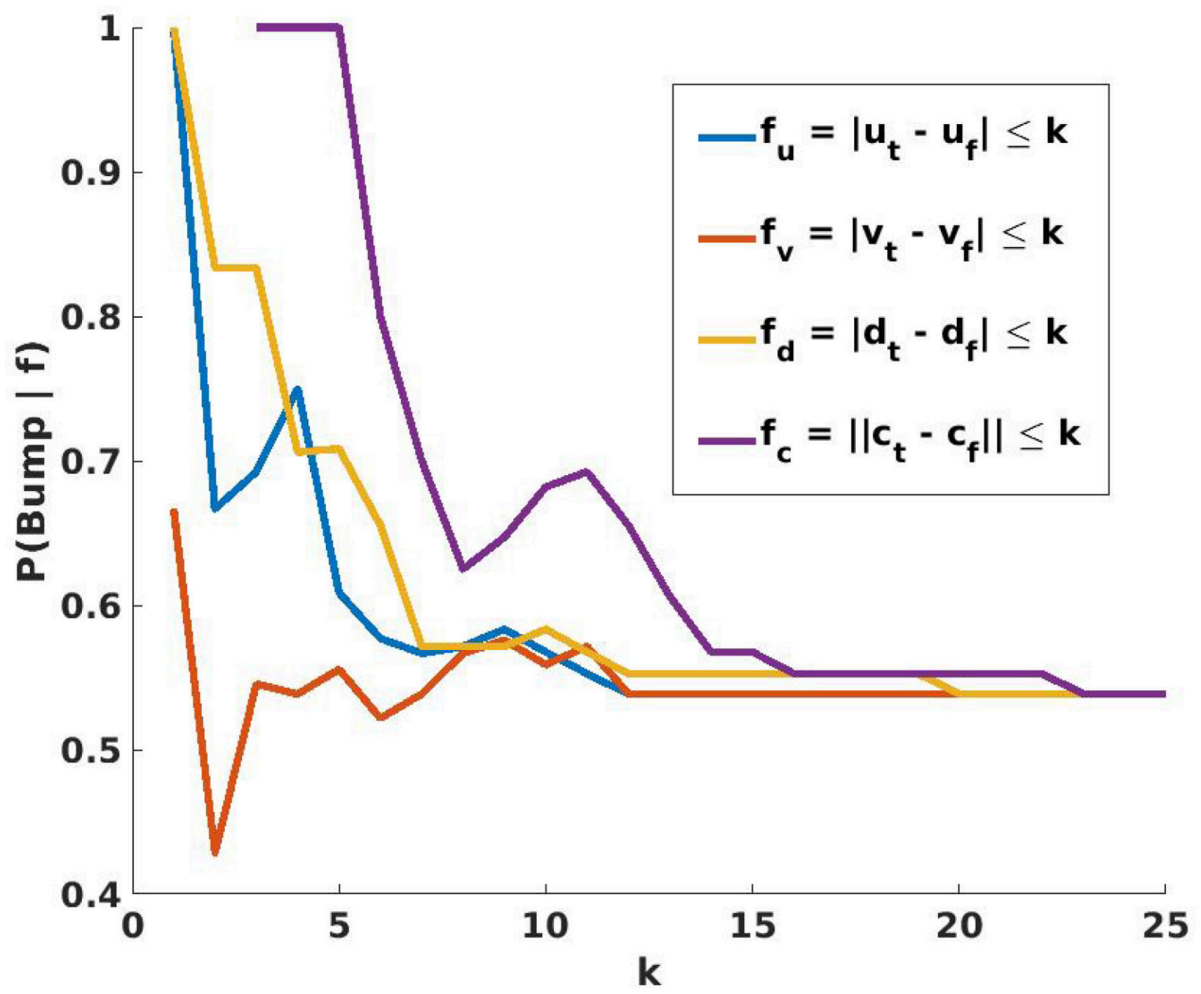
Given percepts for hand and target object, the agent searches for the feature *f* that will maximize the conditional probability *P*(*Bump*∣*f*). Each feature considers the centers of the palm and target in (*u, v, d*) image-space. *f*_*u*_, *f*_*v*_, and *f*_*d*_ evaluate to true if the absolute difference in one coordinate is less than a variable threshold *k*, and *f*_*c*_ is true if the distance between centers is less than *k*. The probabilities shown in this graph are based on the 102 trajectories used previously, and their outcomes. For all values of *k*, *P*(*Bump*∣*f*) is maximized when *f* = *f*_*c*_. The agent therefore selects as *n*_*f*_ the candidate node where the hand is closest to the target object, thereby minimizing *k* and maximizing *P*(*Bump*∣*f*).

Figure 5 shows the results of comparing several different distance measures between the center positions of the hand and of the target object. This result supports the use of the final node candidate with the smallest center to center distance with the target $\left||c^{t}-c_{f}^{p}|\right|$. This result is also included in the comparison in **Figure 7**. Attempting the 40 reaches again, the agent now considers the reach action to be 77.5% reliable, with 31 successes, 7 false negatives, and 2 actual failures to bump the object.

Tabulated results from experiments 3, 5, and 6:

**Table:** 

**Reach reliability given selection method for *n*_*f*_**	
Select random target node *n*_*f*_ from PPS graph (baseline)	20.0%
Select arbitrary candidate node *n*_*f*_	52.5%
Select candidate node *n*_*f*_ with hand center closest to target center	77.5%

This method, for identifying candidate target nodes that increase the probability of bumping a specified block, can be extended to avoid bumping specified blocks.

### 4.3. Interpolating Between PPS Nodes

#### 4.3.1. Methods

Recall that the first improvement to the reach action was to identify a set of candidate final nodes, all nodes where the stored hand representation and the current percept of the target intersect in both the RGB and depth images. Moving to an arbitrary candidate final node instead of a random node from the PPS graph more than doubles the rate at which bumps are successfully caused. However, Figure 5 demonstrates that the success rate for reaches increased as $\left||c_{f}^{p}-c^{t}|\right|$ decreased. Choosing the candidate node nearest to the target object improved the reliability of the reach to 77.5%, but this method is limited by the density of the PPS Graph near the target. Especially in relatively sparse regions of the graph, even the nearest node may not be close enough for a reliable reach. The agent must learn to make small moves off the graph to reach closer to the object than the nearest node.

The PPS graph P is a discrete, sampled approximation to a continuous mapping between the continuous configuration space of the arm, and a continuous space of perceptual images. The full Jacobian model *J*(*q*) relating joint angle changes Δ*q* to changes in hand center coordinates Δ*c* is a nonlinear mapping, dependent on the current state of the arm *q*, a seven-dimensional vector. The full Jacobian is therefore prohibitively difficult for the agent to learn and use. However, P does contain sufficient data for making linear approximations of the relationship between Δ*q* and Δ*c* local to a particular *q*_*i*_ = *q*(*n*_*i*_). This estimate is most accurate near the configuration *q*_*i*_, with increasing error as the distance from *q*_*i*_ increases.

The linear approximation at a node *n*_*i*_ is derived using the neighborhood $N\left(n_{i}\right)\equiv \left\{n_{i^{\prime }}|\exists e_{i,i^{\prime }}\right\}$, the set of all nodes $n_{i^{\prime }}$ connected to *n*_*i*_ by an edge for feasible motion. The local Jacobian estimate Ĵ(*n*_*i*_) considers all edges $e_{i,i^{\prime }}$ such that $n_{i^{\prime }}\in N\left(n_{i}\right)$. Each edge provides an example pair of changes $\Delta q=q_{i^{\prime }}-q_{i}$ and $\Delta c=c_{i^{\prime }}^{p}-c_{i}^{p}$. If there are *m* neighbors, and thus *m* edges, these can be combined as an *m* × 7 matrix Δ*Q* and a *m* × 3 matrix Δ*C*, respectively. Ĵ(*n*_*i*_) is the least squares solution of

(12) $$\begin{array}{r} \Delta QĴ\left(n_{i}\right)=\Delta C. \end{array}$$

For a given change Δ*q* in arm configuration, Δ*q*Ĵ (*n*_*i*_) = Δ*c* gives a local linear estimate of the resulting change Δ*c* in the appearance of the hand. Conversely, given a desired change Δ*c* in the appearance of the hand, the pseudo-inverse ${Ĵ}^{+}\left(n_{i}\right)$ makes it easy to compute the change Δ*q* in arm configuration that will produce that result.

**Figure 6:**
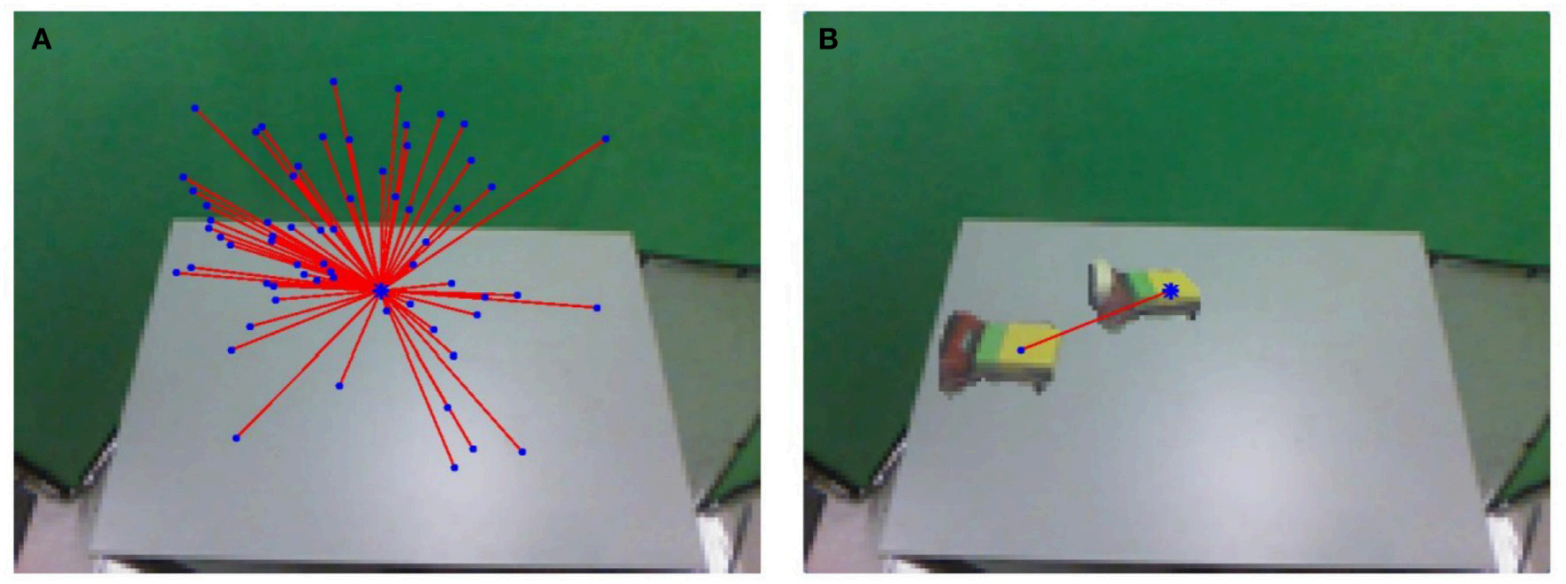
**(A)** The agent considers the graph neighborhood around a node *n*_*i*_ to estimate the change in appearance for small changes in configuration near *n*_*i*_. The predictions will be made by a local Jacobian estimate Ĵ(*n*_*i*_) (see Equation 12). *n*_*i*_ is near the center of P and has a large number of neighbors. Each edge is relatively short in configuration space, where edge feasibility is measured, even though some neighbors appear distant in image space. The furthest neighbors tend to be those where most of the edge length comes from a difference in proximal joint angles that have a larger effect on workspace position. **(B)** The images of the node *n*_*i*_ and one of its neighbors are superimposed with a representation of the edge, drawn between their centers of mass. This example illustrates a change in configuration Δ*q* and the resulting change in center locations Δ*c* along one edge.

Figure 6 shows an example graph neighborhood and a visualization of the information contained in each edge. The resulting Ĵ(*n*_*i*_) is a 7 × 3 matrix where the element at [*row, col*] gives the rate of change for *c*^*col*^ (either the *u*, *v*, or *d* coordinate of the palm's center of mass) for each unit change to *q*^*row*^. A possible adjustment Δ*q* to *q*_*i*_ may be evaluated by determining if the predicted new palm center ${ĉ}_{i}^{p}\equiv c_{i}^{p}+\Delta qĴ\left(n_{i}\right)$ and the palm mask *p*_*i*_ translated by Δ*q*Ĵ(*n*_*i*_) have desirable features. Rotations and shape changes of *p*_*i*_ that will occur during this motion are not modeled, but are typically small.

Where *n*_*f*_ is the final node of the planned trajectory for a *reach*, the agent can use the local Jacobian Ĵ(*n*_*f*_) and its pseudo-inverse ${Ĵ}^{+}\left(n_{f}\right)$ to improve the accuracy of its final motion, and the likelihood of causing a *bump* event.

Where $c_{f}^{p}$ is the center of the palm in the percept in node *n*_*f*_, and *c*^*t*^ is the center of the target object, the desired change in the palm percept is $\Delta c=c^{t}-c_{f}^{p}$. Then the updated final configuration is

(13) $$\begin{array}{r} q_{f}^{*}=q_{f}+\left(c^{t}-c_{f}^{p}\right){Ĵ}^{+}\left(n_{f}\right) \end{array}$$

When the agent moves to the configuration $q_{f}^{*}$, the palm center should be approximately aligned with the target's center. A motion that aligns the centers should increase the size of the intersection, making the action robust to noise, and increasing the likelihood of the resulting *bump* event.

While the ability to make a small move off of the graph to $q_{f}^{*}$ increases the robustness of the reach, it does not eliminate the need for a set of candidate final nodes, or for the decision to use the nearest node to the target as *n*_*f*_. As ${Ĵ}^{+}\left(n_{f}\right)$ is a local estimate, if $\left||c^{t}-c_{f}^{p}|\right|$ is large, the error in the recommended Δ*q* will also tend to be large. Choosing the nearest candidate *n*_*f*_ minimizes the factor by which natural errors in ${Ĵ}^{+}\left(n_{f}\right)$ will be multiplied, giving the best accuracy for the final position of the reach. Adding the use of the inverse local Jacobian gives the final reaching procedure below.

#### 4.3.2. Experiment 7: Reaching to Target Adjusted by Local Jacobian

The final improvement in the *reach* action starts with the trajectory planned to the closest candidate node *n*_*f*_ to the target object. The configuration *q*_*f*_ in that node is then adjusted according to the local Jacobian for the neighborhood of *n*_*f*_. The final motion in the trajectory then goes to $q_{f}^{*}$, rather than *q*_*f*_. In effect, the PPS graph supports a local linear approximation to the full Jacobian over the continuous configuration space, based in the neighborhood of each node.

This improved policy is evaluated the same way as Experiments 3, 5, and 6.

#### 4.3.3. Experiment 7 Results

**Figure 7:**
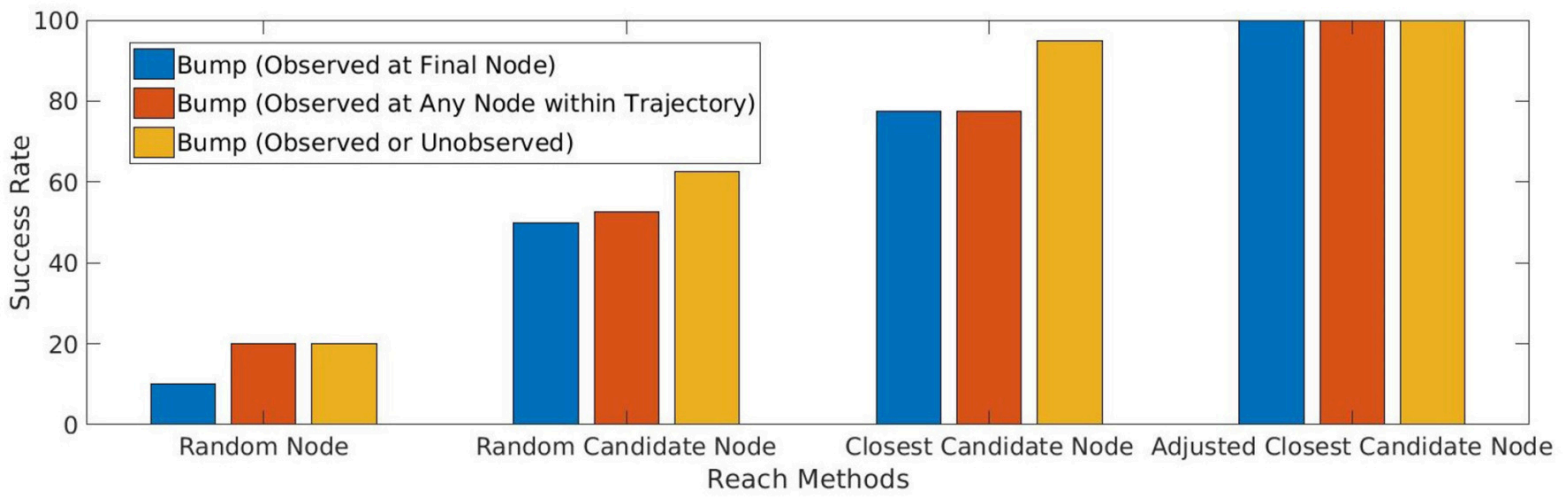
Reliability of the agent's action to reach and bump a single target object by following a trajectory to a selected target node. The four groups represent (1) randomly selected target node; (2) random selection from among candidate nodes with non-empty image intersections; (3) select closest among candidate nodes; (4) adjust node with local Jacobian to best match target object. Within each group, the bars represent different criteria for success: (l) observed bump at final node, which measures the agent's ability to cause bumps intentionally and efficiently; (m) observed bump anywhere in the trajectory, which identifies bumps that can be learned from; (r) any bump, observed or unobserved, which measures ground truth of bump occurrence.

Using this procedure on the training set of target placements, the agent perceives bumps at the final node of all 40 trajectories. This 100% result demonstrates that the reach action has become reliable, and is a significant improvement from the previous methods shown in Figure 7.

**Table:** 

**Reach reliability given selection method for *n*_*f*_**	
Select random target node *n*_*f*_ from PPS graph	20.0%
Select arbitrary candidate node *n*_*f*_	52.5%
Select candidate node *n*_*f*_ with hand center closest to target center	77.5%
Adjust target away from *n*_*f*_ using local Jacobian	100.0%

## 5. Learning a Reliable Grasp Action

In our model, after the intrinsic motivation pattern has resulted in a reliable reach action, the pattern may be applied a second time to learn a grasp action. As the reach action toward a target object becomes more reliable, the result of causing a quasi-static change in the image of that object becomes more typical. However, there is an unusual result: during the interaction with the target object, the hand may reflexively close, providing sensorimotor experience with attempted and successful “accidental grasps.”

Driven by intrinsic motivation, the grasp action becomes more reliable, toward becoming sufficient to serve as part of a pick and place operation in high level planning. In this case, additional requirements may be learned in a more flexible order, so we present the learning stages of our agent according to the order in which it considered the concepts. The agent must begin with the Palmar reflex to observe the unusual results of a reliable reach action without consciously closing the hand with correct timing. Our agent then learned: how to most reliably set the gripper's aperture during the grasp approach, how to best align the hand, target, and final motion, and how to preshape the hand by orienting the wrist. Each stage is presented with a Methods-Experiments-Results description.

### 5.1. Reaching With an Innate Palmar Reflex

#### 5.1.1. Methods

Human infants possess the *Palmar reflex*, which closes the hand as a response to contact of an object to the palm. Our work assumes that the Palmar reflex is innate and persistent during at least early stages of learning to grasp. Within our framework, the primary importance of this reflex is to enable the observation of accidental grasps as an unusual event while reaching. While the closing of the hand is unconscious, the agent learns the motor commands and sensations of closing the hand.

When conditions are correct, the Palmar reflex causes an accidental *grasp*, where the object is held tightly in the hand and becomes a temporary part of the self. This gives a much greater level of control over the pose of the object, as it can be manipulated with the agent's learned scheme for moving the hand until the relationship ends with an *ungrasp*, opening the fingers to release the object. The variety of outcomes possible with the level of control a grasp provides imply a high potential reward from learning to predict the outcomes and actions to cause them, but it is also the case that grasps occur too rarely to learn immediately after learning to reach. Without enough examples, learning the conditions for a grasp may prove too difficult, leading to a modest rate of improvement and a low reward. In our model, the agent focuses next on an intermediate rare event.

The activation of the Palmar reflex is such an event that may be observed as an unusual result of successful reaches. When the hand's final approach to the target meets all necessary conditions of openness, alignment, and orientation, the target object passes between the grippers in a way that activates the simulated Palmar reflex, and the gripper fingers close. The openness of the grippers is a degree of freedom for the robot's motion, and is continually sensed by proprioception. As a result, accurate detection of when the Palmar reflex has been triggered does not rely on the visual percept, and can be observed in a rapid decrease of openness to a new fixed point.

The closing of the grippers, either by reflex or conscious decision, is necessary for the agent to gain a higher level of control over the object with a *grasp*. In some cases, the initial interaction between the hand and object does not lead to the grippers closing around the object, and the attempt to gain control fails immediately. We refer to this event as a *Palmar bump*, as it often involves knocking away the object before the grippers can close on it. Like other bumps, this is a quasi-static change with an observably low IOU value between masks, and it is the result of a successful reach. While the Palmar bump is not a successful grasp, it serves as a useful near-miss example, promoting use of the conditions that allowed the reflex to trigger in future grasp attempts.

**Figure 8:**
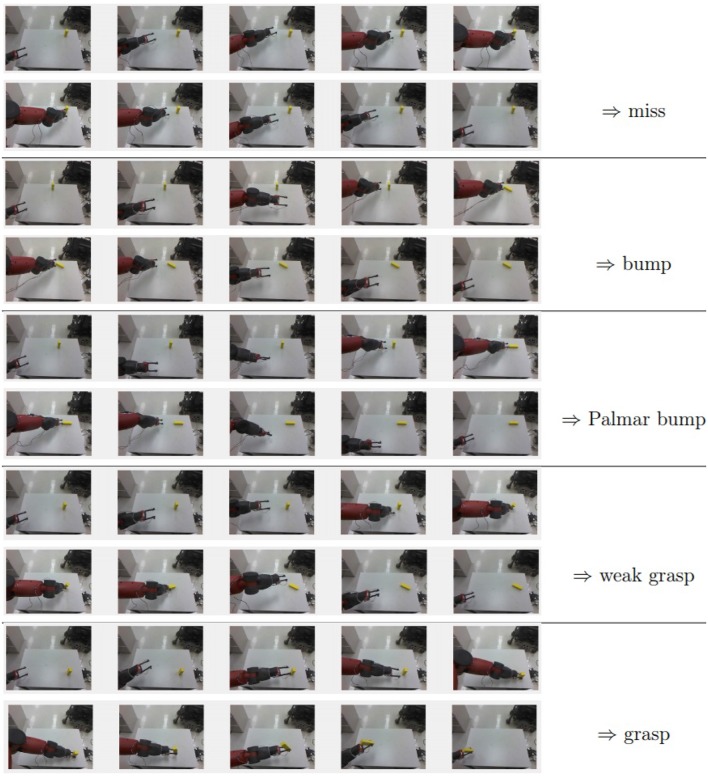
The agent's RGB percepts during attempted grasp trajectories. Images for the forward portion toward *n*_*f*_ are shown in the first of each pair of rows, and images for the portion to return to *n*_*h*_ are shown in the second rows. Images for some nodes in the middle of trajectories with more than five nodes have been omitted. The agent classifies the result of the grasp attempt by observing the state of the target object during the trajectory. In all cases but *miss*, there is a substantial change between the first and last observations, and the trajectory is a successful reach. In all other cases these observations should be significantly different, and the reach component of the grasp was successful. Further classification depends on the state throughout the return trajectory and if the Palmar reflex was activated, as discussed in section 5.1.1. Only the result of the final example is considered to be a successful grasp.

When a *grasp* occurs, the activation of the Palmar reflex is followed by the object shifting from its initial quasi-static state to a new dynamic state. Now held between the gripper fingers, the object begins to follow the hand with continued motion correlated with the motion of the hand. The agent can identify this corresponding motion by comparing masks and depth ranges during the return trajectory. A grasp is successful if and only if the stored masks and depth ranges for each node of the trajectory intersect with those of the target object in the visual percepts during the return to the home node. Note that the full hand masks and depth ranges are used since the gripper fingers, once closed, may obscure the portion of the object in the palm region. If all nodes of the trajectory have an empty mask or depth range intersection, control was never gained and the result is a Palmar bump. If at least one node fails the intersection check, but not all nodes, the grasp is considered to be a *weak grasp*. Here the grasp was initiated, but due to a loose or poor placement, did not persist through the return trajectory. Note that the loss of control of the object in a weak grasp does not involve an opening of the grippers, as an intentional *ungrasp* action would. Figure 8 provides an example of the agent's visual percepts of a trajectory that produced each type of result.

Since the Palmar bump and weak grasp cases fail to gain or maintain control of the object, both are successful reaches but failed grasps. By considering both situations to be failures, the successful grasps that emerge from this learning process are more likely to facilitate subsequent learning of higher order actions that require a grasp. However, Palmar bumps, weak grasps and grasps share the sensed result of reflexively closing the hand, and may be assumed to share similar preconditions as well. Until a sufficient number of successful grasps are observed, the agent will draw information from all cases where the Palmar reflex was activated to learn to grasp.

#### 5.1.2. Experiment 8: Monitoring the Palmar Reflex During Reaching

We first attached a break-beam sensor between the tips of the Baxter robot's parallel gripper fingers to provide the agent with a simulated innate Palmar reflex. Then our agent repeated all trials of Experiments 3, 5, 6, and 7 in section 4, using the same target placements and planned trajectories. For each trial, the agent records if the Palmar reflex was activated, and which category of result (grasp, weak grasp, Palmar bump, bump, or miss) it observed.

#### 5.1.3. Experiment 8 Results

It is clear that learning to reach more reliably and with greater precision allows more Palmar reflex activations and grasps to occur. With the random trajectories of Experiment 3, one of 40 activated the Palmar reflex, and this was a successful grasp. Using the final reaching method of Experiment 7, the agent observed that the Palmar reflex was activated in 12 out of the 40 trials. Of these 12, 5 were successful grasp trajectories. These provide a baseline reliability of grasping with random motion trajectories (2.5%) and of grasping with a reliable reach trajectory (12.5%). These results and those for intermediate reach methods are tabulated below, and also shown alongside the rest of the results for this section numerically in **Figure 11** and spatially in **Figure 12**.

Tabulated results from experiment 8:

**Table:** 

**Results**	**Grasp: Successful**	**Failed**			
	**Palmar Reflex: Activated**			**No Activation**	
	**Reach: Successful**				**Failed**
		Weak	Palmar		
	Grasp	Grasp	Bump	Bump	Miss
Experiment 8 (3)	2.5%	0%	0%	17.5%	80.0%
Experiment 8 (5)	2.5%	0%	7.5%	42.5%	47.5%
Experiment 8 (6)	5.0%	0%	12.5%	60.0%	22.5%
Experiment 8 (7)	12.5%	0%	17.5%	70.0%	0%

### 5.2. Initiating Grasps With the Gripper Fully Open

#### 5.2.1. Methods

While exploring PPS and performing reaches, the agent is motivated to keep the hand fully open (*a* = 100). This presents the largest silhouette of the hand to keep in view, as desired, and the full extension allows for more interactions with objects when the extremities collide with them. As the PPS Graph was created, this setting also allowed a brightly colored block to be placed spanning the full width of the grippers, simplifying visual tracking of the “palm.”

With the new event of a Palmar reflex activation during the interaction, the agent may choose to investigate its degrees of freedom. Each of the joint angles in *q* have an understood role in the placement of the hand, but *a* does not appear to significantly affect the location of the hand's center of mass and does not differentiate graph nodes. This allows it to be freely modified to investigate its influence on the frequency of Palmar reflex activations.

#### 5.2.2. Experiment 9: Which Gripper Aperture Setting Is Most Reliable?

While it is intuitively desirable for the agent to approach targets with the grippers open for a Palmar bump or grasp, the agent does not yet have sufficient data to reach this conclusion. This is gathered by repeating the trajectories of Experiment 6, the final reaching method, with the Palmar reflex active and each gripper aperture of 0, 25, 50, and 75% open. These four sets of results can be compared with those for the fully open gripper that were already obtained in Experiment 7.

#### 5.2.3. Experiment 9 Results

**Figure 9:**
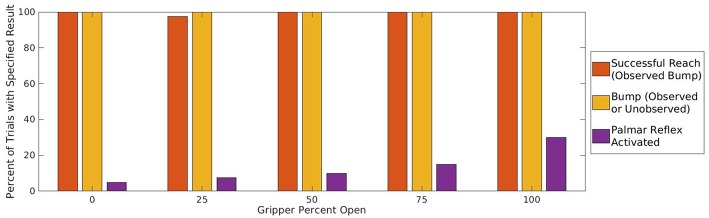
The portion of attempted reach trajectories that produce observed bumps (orange), ground truth bumps (yellow), and Palmar bumps, or bumps which also trigger the Palmar reflex (purple) for varying gripper apertures *a*. The high reliability of the reach action is independent of *a*, indicating it could be learned and executed with any setting. By contrast, triggering the Palmar reflex is much more likely as *a* increases, and is learned as a prerequisite for the Palmar bump event and later for the grasp action.

Two conclusions may be drawn from the results of this experiment, which are visualized in Figure 9. First, it is clear that the probability of activating the Palmar reflex increases with the openness *a* of the gripper during the approach. As *a* decreases, the opening of the hand narrows, and the object is less likely to pass inside with an approach of equal precision, so there are less activations. Once *a* is sufficiently low that the object cannot fit in the hand, the Palmar reflex never triggers. The agent will continue using the fully open setting *a* = 100 in future attempts to maximize its expected success rate.

Second, we see that the openness of the gripper has almost no affect the probability of a bump. In fact, only one trial was perceived to fail with any setting, and this was a false negative. We claim that this demonstrates the agent could have learned the reach action with the same process and ending reliability for any gripper setting, and at that point would learn to prefer 100% open. It is therefore not necessary for our model to assume any initial setting *a* for the gripper opening while learning to reach.

### 5.3. Planning the Approach With Cosine Similarity Features

#### 5.3.1. Methods

When reaching, it is important that the candidate final nodes satisfying Equation (11) are identified, and *n*_*f*_ is chosen to minimize $\left||c^{t}-c_{f}^{p}|\right|$. To plan reaches that activate the Palmar reflex, additional features are needed to ensure not only that the final position is correct, but also that the hand orientation and the direction of final motion are suitable. These must be compatible during the approach, and must also be effective for the current target object. To learn to use satisfactory relationships between these vectors, the agent constructs this set of vectors using information from its stored and current visual percepts:

(14) $$\begin{array}{l} \begin{array}{l} \text{gripper vectors: }\text{pointing outward, near parallel to the gripper fingers.}\\ {\;\vec{g}}_{p}\equiv \text{drawn from }c_{p}^{h}\text{ through }c_{p}^{p}\\ \;{\vec{g}}_{f}\equiv \text{drawn from }c_{f}^{h}\text{ through }c_{f}^{p}\\ \text{motion directions: }\text{direction of motion along an edge or toward a target}\\ {\vec{m}}_{p,f}\equiv \text{the direction of the edge-based final motion from }c_{p}^{p}\text{ to }c_{f}^{p}\\ {\vec{m}}_{p,t}\equiv \text{the direction of the modified final motion from }c_{p}^{p}\text{ to }c^{t}\\ {\vec{m}}_{f,t}\equiv \text{the direction of displacement from }c_{f}^{p}\text{ to }c^{t}\\ \text{object orientation: }\text{the perceived major axis of the target object}\\ \;\vec{o}\equiv \text{drawn along the major axis of }t\text{.} \end{array} \end{array}$$

The agent learns cosine similarity criteria for the vectors of final motions that most reliably cause Palmar reflex activations in Experiment 10. In Experiment 11, the agent plans trajectories with final motions that satisfy this criteria to improve the reliability of Palmar reflex activations and grasps.

#### 5.3.2. Experiments

##### 5.3.2.1. Experiment 10: Learning reliable cosine similarities

To discover the best relationship between these vectors for repeating the Palmar reflex activation event, the agent uses the data from repeating the final reach trajectories of Experiment 7 in Experiment 8 with the Palmar reflex enabled. For each trajectory, it considers the cosine similarity $C\left({\vec{v}}_{1},{\vec{v}}_{2}\right)$ of each pair ${\vec{v}}_{1},{\vec{v}}_{2}\in \left\{{\vec{g}}_{p},{\vec{g}}_{f},{\vec{m}}_{p,f},{\vec{m}}_{p,t},{\vec{m}}_{f,t},\vec{o}\right\}$ and results. The cosine similarities are discretized to the nearest value in {−1, −0.5, 0, 0.5, 1}. The rate of Palmar reflex activations is observed for trajectories grouped by their discretized *C* values.

##### 5.3.2.2. Experiment 11: Planning well-aligned final motions

**Figure 10:**
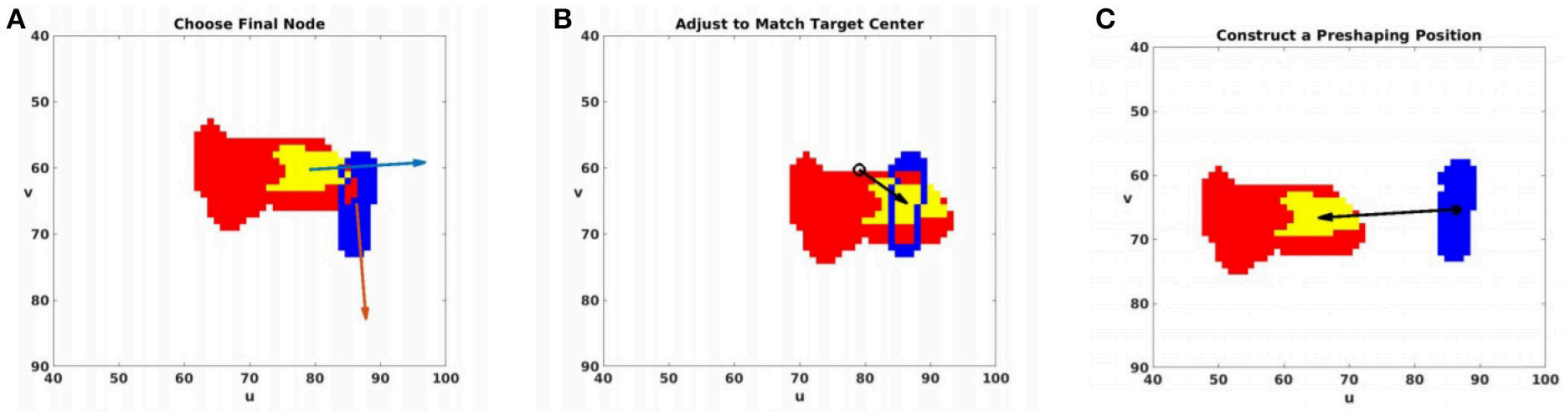
The agent plans modifications to the end of the trajectory, and defines a preshaping configuration $q_{p}^{*}$. Human intuition and the agent's learning recognize that all vectors describing gripper direction and the direction of motion should be near parallel, with all of these vectors near perpendicular to the target's major axis. The agent plans a final motion with these features in three steps: **(A)** The agent chooses *n*_*f*_ from the candidate final nodes (Equation 11) to minimize $C\left({\vec{g}}_{f},\vec{o}\right)$. This image displays the palm mask (yellow) and hand mask (red) for the chosen *n*_*f*_, along with the target mask (blue). A blue outline is used to show the boundary of the intersection between the hand and target. ${\vec{g}}_{f}$ and $\vec{o}$ are displayed in light blue and orange, respectively. **(B)** The agent uses ${Ĵ}^{+}\left(n_{f}\right)$ to estimate the change in joint angles necessary to cause the image-space translation shown here. This translation improves the approach accuracy by aligning $c_{f}^{p}$ with *c*^*t*^ by moving to $q_{f}^{*}$. **(C)** The agent constructs $q_{p}^{*}$ by Equation (15), which is predicted to have masks translated as shown from those for $q_{f}^{*}$. A final motion from $q_{p}^{*}$ to $q_{f}^{*}$ has aligned gripper and motion vectors.

The agent uses the results of Experiment 10 to plan the next set of trajectories to interact with the target. At this time, the agent does not have the ability to change any ${\vec{g}}_{i}$ to a particular direction to be perpendicular to $\vec{o}$. Therefore, instead of the nearest candidate final node, *n*_*f*_ is selected from the candidates such that $\left|C\left({\vec{g}}_{f},\vec{o}\right)\right|$ is minimized. As before, ${Ĵ}^{+}\left(n_{f}\right)$ is computed and used to modify the final configuration to a more reliable $q_{f}^{*}$ by Equation (13). The agent may apply ${Ĵ}^{+}\left(n_{f}\right)$ again to create a preshaping position, a copy of the final position translated in the direction of $-{\vec{g}}_{f}$. This image-space translation has a magnitude of 21, the mean length of the final motion for all Palmar bumps and grasps previously observed. The preshaping position has configuration

(15) $$\begin{array}{r} q_{p}^{*}=q_{f}^{*}+21\left(-{\vec{g}}_{f}/||{\vec{g}}_{f}||\right) \end{array}$$

and will replace *q*_*p*_. With this use of ${Ĵ}^{+}\left(n_{f}\right)$, it is expected that ${\vec{g}}_{p}\approx {\vec{g}}_{f}$, and the motion from $q_{p}^{*}$ to $q_{f}^{*}$ should be in the direction of ${\vec{g}}_{f}$, opposite of the translation. In place of ${\vec{m}}_{p,f}$, ${\vec{m}}_{p,t}$, and ${\vec{m}}_{f,t}$, the direction of this motion is parallel to the gripper vector and near perpendicular to the target major axis. The three steps of choosing *n*_*f*_, adjusting to $q_{f}^{*}$ to match centers with the target, and translating to create a well-aligned preshaping position with $q_{p}^{*}$ are visualized in Figure 10.

The agent must plan a trajectory that ends with this approach. $q_{p}^{*}$ is not stored in P, so to find a feasible path to $q_{p}^{*}$, the agent first identifies the nearest node *n*_*n*_ ∈ P that minimizes $\left||q_{p}^{*}-q_{n}|\right|$. A graph search then yields the shortest path from the home node to *n*_*n*_. After visiting *n*_*n*_, the arm will be moved from *q*_*n*_ to $q_{p}^{*}$, and then make the final motion to $q_{f}^{*}$ to complete the trajectory.

The reliability of the grasp action using this method for planning trajectories with aligned final motions is evaluated using the same layout of target placements as Experiment 7, with the Palmar reflex enabled as in Experiment 8. The agent also continues to record the frequency of all types of Palmar reflex activations.

#### 5.3.3. Results

##### 5.3.3.1. Experiment 10 results

When ${\vec{v}}_{1}\neq \vec{o}$ and ${\vec{v}}_{2}\neq \vec{o}$, the highest rate of Palmar reflex activations occurs in the $C\left({\vec{v}}_{1},{\vec{v}}_{2}\right)\approx 1$ group. For any ${\vec{v}}_{1}\neq \vec{o}$, the trajectories where $C\left({\vec{v}}_{1},\vec{o}\right)\approx 0$ have the highest rate. The agent concludes that the ideal approach for the Palmar reflex activation event should use matching directions for all vectors describing the motion and orientation of the hand, $\left\{{\vec{g}}_{p},{\vec{g}}_{f},{\vec{m}}_{p,f},{\vec{m}}_{p,t},{\vec{m}}_{f,t}\right\}$, and all of these parallel vectors should be perpendicular to the target's major axis $\vec{o}$.

##### 5.3.3.2. Experiment 11 results

**Figure 11:**
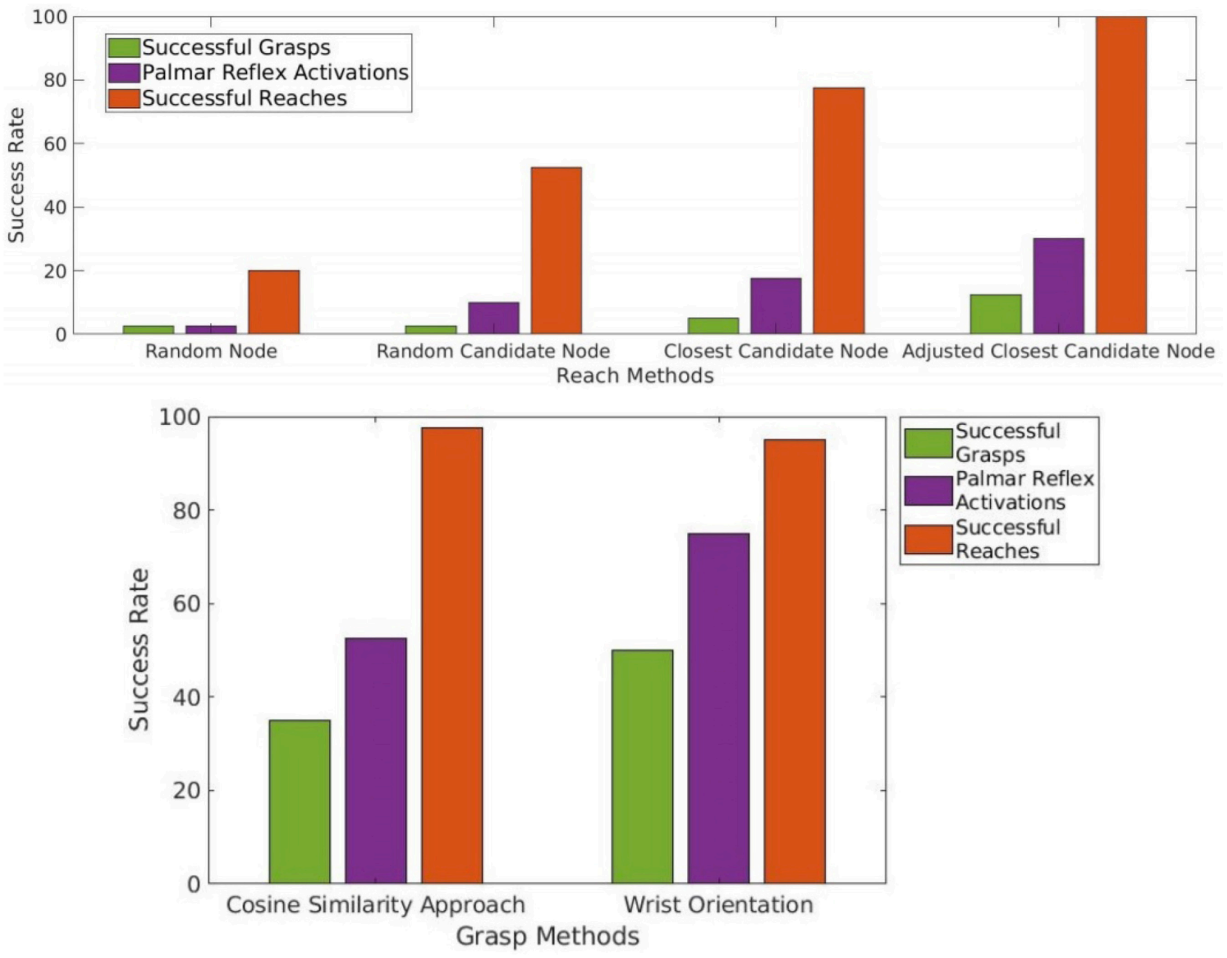
The top plot presents the overall results from the reaching methods as a baseline for the grasp action as found in Experiment 8. The final reach method, Adjusted Closest Candidate Node (Experiment 7), is always successful at reaching, but within these interactions only 12.5% are fully successful though accidental grasps. By considering additional features, the grasp methods in the bottom plot all achieve more than double this success rate for grasping with only modest decreases in reach reliability. The Cosine Similarity Approach Method (Experiment 11) aims to increase the number of Palmar Bumps, with *n*_*f*_ chosen from the candidates such that $\left|C\left({\vec{g}}_{f},\vec{o}\right)\right|$ is minimized and with *n*_*p*_ replaced by a preshaping position so that all other cosine similarities are 1. Approaching with a motion parallel to ${\vec{g}}_{f}$ and perpendicular to $\vec{o}$ also increases the number of successful grasps. The Wrist Orientation Method (Experiment 12) further adds a technique to copy the most distal degree of freedom *q*_7_ used at the nearest configuration to previously succeed, converting more bumps into Palmar bumps and grasps.

**Figure 12:**
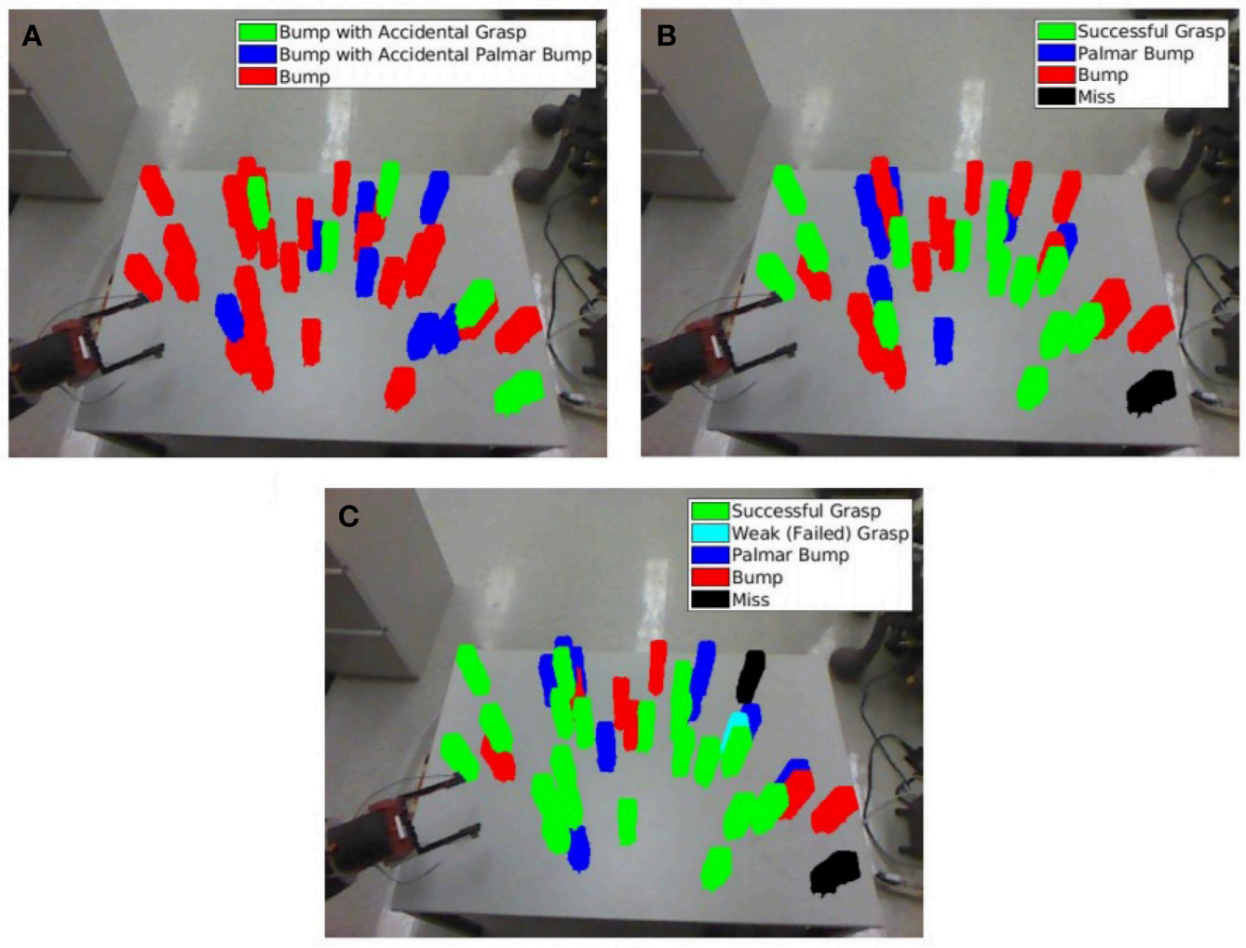
Spatial representations of the results of three methods for the agent's learned reach and grasp actions. Each shows a superposition of all placements of the single target object, colored according to the result of the agent's attempt to repeat an unusual event by executing a motion trajectory. **(A)** Experiment 8 uses the final reaching trajectories of section 4.3 to successfully repeat the bump event for all target placements. Twelve of these reaches accidentally trigger the Palmar reflex, five of which become early examples of grasps. **(B)** Using cosine similarity features in Experiment 11 (section 5.3), the agent modifies the final approach so that this motion causes significantly more Palmar bumps and more grasps are also observed. **(C)** In results from experiment 12, the agent grasped from additional placements by changing the angle of the most distal joint, w2. The wrist orientation is copied from the final configuration of a trajectory that succeeded for a nearby placement (section 5.4). The use of nearest neighbors applies best very close to existing successes, so most improvements can be observed in these areas.

Using trajectories planned in this manner, 39 of 40 reaches are successfully completed and 21 of these activate the Palmar reflex. 14 of these activations result in a grasp. By choosing the best aligned candidate final node instead of the closest candidate node and then adjusting the entire final motion to match its gripper vector, the reliability of grasping is nearly tripled to 35%. Figures 11, 12 provide additional comparisons with results from other learning stages.

Tabulated Results from experiments 8 and 11:

**Table:** 

**Results**	**Grasp: Successful**	**Failed**			
	**Palmar Reflex: Activated**			**No Activation**	
	**Reach: Successful**				**Failed**
	Grasp	Weak Grasp	Palmar Bump	Bump	Miss
Experiment 8 (3)	2.5%	0%	0%	17.5%	80.0%
Experiment 8 (5)	2.5%	0%	7.5%	42.5%	47.5%
Experiment 8 (6)	5.0%	0%	12.5%	60.0%	22.5%
Experiment 8 (7)	12.5%	0%	17.5%	70.0%	0%
Experiment 11	35.0%	0%	17.5%	45.0%	2.5%

### 5.4. Orienting the Grippers With the Wrist

#### 5.4.1. Methods

For our Baxter robot, the joint angle setting *q*^7^, which controls the most distal twist joint, “wrist 2” or *w*2, affects only a small portion of the wrist with a roll of the hand relative to the axis of the forearm without changing this axis. This alters the orientation and perceived shape of the gripper opening, but leaves the position largely unchanged. The primary modification is to the plane in which the gripper fingers open and close. Adjusting this is analogous to a human's preshaping techniques to ready the hand for grasping an object, though simpler, as there are fewer ways to configure parallel grippers than an anthropomorphic hand. For a grasp to be successful, the cross section of the object in the gripper plane must be smaller than the space between the grippers. Additionally, the angle at which the plane and the object meet must not be so steep as to squeeze the object out of the grip. Intuitively, the most reliable grasp approach rotates *w*2 so that the gripper plane is perpendicular to the target object's major axis.

#### 5.4.2. Experiment 12: Copying Successful Wrist Settings

Without intuition for the correct orientation, the agent must find another criteria for predicting the wrist orientation that will be most reliable. By this time, the agent has observed that, like the gripper aperture *a*, *q*^7^ does not have a significant impact on the hand's location in the image. This allows the agent to consider modifying *q*^7^ without considering the graph nodes visited to change. In the same way, these changes do not conflict with the learned requirements for reaching or the previous grasping method of choosing *n*_*f*_ such that ${\vec{g}}_{f}$ and $\vec{o}$ are approximately perpendicular. In order to avoid new failures from introducing large, sudden rotations of the hand near the target, when a new *q*^7^ is chosen it will be used instead of the stored *q*^7^ value of all nodes in the trajectory *n*_*T*_*j*__.

To begin, the agent repeats each successful grasp, with a linear search over values of *q*^7^ to identify the longest continuous range where the attempt still succeeds. The center of this range will be saved as the ideal *q*^7^ value for this example grasp. The agent will then retry each trajectory from Experiment 11. For each of these grasp attempts, the adjusted final configuration $q_{f}^{*}$ is computed by Equation (13), as before. Using the Euclidean distance between all other joint angles, $\langle q_{f}^{1},\ldots ,q_{f}^{6}\rangle$, the nearest neighbor example grasp is found for the current trial. The grasp is attempted with the ideal *q*^7^ value from this example and all other angles unchanged.

#### 5.4.3. Experiment 12 Results

Over the same set of 40 object placements from previous experiments, this technique increases the number of Palmar reflex activations (Palmar bumps, weak grasps, and grasps) to 30 (75%), and grasps to 20 (50%), as shown in Figures 11, 12. These increases come at the cost of one bump, where the target is now missed because the rotation of the hand prevents a collision that used to narrowly occur. In principle, any time new successes are achieved, they can be treated as new example grasps with ideal *q*^7^ values to consider for trials with nearby target placements, allowing for further improvements to the success rate. However, in this training set only two still unsuccessful grasp attempts have different nearest neighbor examples than previously, and neither changes to a success with the new *q*^7^ value. Iterations of using new nearest neighbors therefore end, but may be returned to in future work once more examples are available.

Tabulated Results from experiments 8, 11, and 12:

**Table:** 

**Results**	**Grasp: Successful**	**Failed**			
	**Palmar Reflex: Activated**			**No Activation**	
	**Reach: Successful**				**Failed**
	Grasp	Weak Grasp	Palmar Bump	Bump	Miss
Experiment 8 (3)	2.5%	0%	0%	17.5%	80.0%
Experiment 8 (5)	2.5%	0%	7.5%	42.5%	47.5%
Experiment 8 (6)	5.0%	0%	12.5%	60.0%	22.5%
Experiment 8 (7)	12.5%	0%	17.5%	70.0%	0%
Experiment 11	35.0%	0%	17.5%	45.0%	2.5%
Experiment 12	50.0%	2.5%	22.5%	20.0%	5.0%

## 6. Conclusions

We have demonstrated a computational model of an embodied learning agent, implemented on a physical Baxter robot, exploring its sensorimotor space without explicit guidance or feedback, constructing a representation of the robot's peripersonal space (the PPS graph), including a mapping between the proprioceptive sensor and the visual sensor.

We make use of a specific form of intrinsic motivation. After learning the typical result of an action, and identifying an unusual outcome, the agent is motivated to learn the conditions that make the unusual outcome reliable. We apply this process once to learn reliable reaching, and again to learn (relatively) reliable grasping.

This work makes several contributions to developmental learning:

### 6.1. The Peripersonal Space (PPS) Graph

By unguided exploration of the proprioceptive and visual spaces, and without prior knowledge of the structure or dimensionality of either space, the learning agent can construct a graph-structured skeleton (the PPS Graph) that enables manipulator motion planning by finding and following paths within the graph. The graph representation requires only limited knowledge of the attributes of the nodes, and no knowledge of the dimensionality of the embedding space.

### 6.2. Learning Reliable Reaching

By learning conditions to make a rare action (i.e., reaching to cause a bump of a block) reliable, the agent learns a criterion on perceptual images (stored and current) that allows it to select a suitable target node in the PPS Graph. Motion to that target node accomplishes a reliable reach. The PPS Graph representation accounts for reaching in a way that matches striking qualitative properties of early human infant reaching: jerky motion, and independence from vision of the hand.

By interpreting the target node and its neighborhood as a sample from a continuous space, the agent can approximate the local Jacobian of the hand pose in perceptual space with respect to the joint angles. This allows it to adjust the trajectory to make reaching more reliable.

### 6.3. Learning Reliable Grasping

At this point, reaching reliably displaces the target block. Occasionally, instead of quasi-statically displacing the block, the block continues to move, to follow the subsequent motion of the hand. Making this result reliable requires several distinct conditions. The innate Palmar reflex makes these rare events common enough to learn from. Conditions on gripper opening, wrist orientation, and approach direction can all be learned based on positive feedback from the unusual block motion.

### 6.4. Future Research Directions

Our current model is very simple, yet it supports learning of reliable reaching and grasping. We hypothesize that an improved dynamical model of hand motion will better explain early jerky motion. We also hypothesize that progress toward smooth, directed, adult reaching will build on approximated interpolation methods exploiting information in the PPS graph, such as the local Jacobian. Finally, we expect to be able to model improvements in the visual system, allowing observations of the size and shape of the target object to influence pre-shaping of the hand.

### 6.5. Significance for Developmental Learning

There have been recent impressive results from unguided end-to-end learning of multiple games (Silver et al., 2017, 2018). While these results are very exciting, some limitations come from the need for vast amounts of training experience, and the lack of transparency and explainability of the learned knowledge.

We hope that our work on reaching and grasping in peripersonal space can illuminate the kinds of intermediate states that a developmental learner goes through. Those intermediate states make the structure of the knowledge more comprehensible, and the learning stages between them more efficient. Combining the strengths of these approaches could be important.

## Author Contributions

BK contributed the initial conception. BK and JJ collaborated on the development of the model and the design of the study, analyzing the data, wrote sections of the manuscript, and both contributed to manuscript revision, and read and approved the submitted version. JJ created the robot implementation, carried out the experiments, and collected the data.

### Conflict of Interest Statement

The authors declare that the research was conducted in the absence of any commercial or financial relationships that could be construed as a potential conflict of interest.

## Footnotes

^1^Strictly speaking, a graph P = 〈N, E〉 consists of two sets, one for nodes and one for edges. For notational simplicity, we will use *n*_*i*_ ∈ P and *e*_*ij*_ ∈ P as abbreviations for *n*_*i*_ ∈ N(P) and *e*_*ij*_ ∈ E(P).

^2^We use the word “palm” for this region because of its functional (though not anatomical) similarity to the human palm, especially as the site of the Palmar reflex (Futagi et al., 2012).
